# A critical realist synthesis of cross-disciplinary health policy and systems research: defining characteristic features, developing an evaluation framework and identifying challenges

**DOI:** 10.1186/s12961-020-00556-2

**Published:** 2020-07-14

**Authors:** Gordon Dugle, Joseph Kwame Wulifan, John Paul Tanyeh, Wilm Quentin

**Affiliations:** 1 Department of Management Studies, School of Business and Law, University for Development Studies, Box UPW 36, Wa Campus, Wa, Ghana; 2 Nottingham University Business School, Jubilee Campus, Nottingham, NG8 1BB UK; 3 Department of Healthcare Management, TU, Berlin, Germany; 4 European Observatory on Health Systems and Policies, Berlin, Germany

**Keywords:** Health policy and systems research, Cross-disciplinary research, Multidisciplinary research, Interdisciplinary research, Transdisciplinary research, Critical realist synthesis

## Abstract

**Background:**

Health policy and systems research (HPSR) is an inherently cross-disciplinary field of investigation. However, conflicting conceptualisations about inter-, multi- and transdisciplinary research have contributed to confusion about the characteristics of cross-disciplinary approaches in HPSR. This review was conducted to (1) define the characteristic features of context–mechanism–outcome (CMO) configurations in cross-disciplinary HPSR, (2) develop criteria for evaluating cross-disciplinarity and (3) synthesise emerging challenges of the approach.

**Method:**

The paper is a critical realist synthesis conducted in three phases, as follows: (1) scoping the literature, (2) searching for and screening the evidence, and (3) extracting and synthesising the evidence. Five databases, namely the International Bibliography of the Social Sciences and Web of Science, PubMed central, Embase and CINHAL, and reference lists of studies that qualified for inclusion in the review were searched. The search covered peer-reviewed original research, reviews, commentary papers, and institutional or government reports published in English between January 1998 and January 2020.

**Results:**

A total of 7792 titles were identified in the online search and 137 publications, comprising pilot studies as well as anecdotal and empirical literature were selected for the final review. The review draws attention to the fact that cross-disciplinary HPSR is not defined by individual characteristics but by the combination of a particular type of research question and setting (context), a specific way of researchers working together (mechanism), and research output (outcome) that is superior to what could be achieved under a monodisciplinary approach. This CMO framework also informs the criteria for assessing whether a given HPSR is truly cross-disciplinary. The challenges of cross-disciplinary HPSR and their accompanying coping mechanisms were also found to be context driven, originating mainly from conceptual disagreements, institutional restrictions, communication and information management challenges, coordination problems, and resource limitations.

**Conclusion:**

These findings have important implications. First, the CMO framework of cross-disciplinary HPSR can provide guidance for researchers engaging in new projects and for policy-makers using their findings. Second, the proposed criteria for evaluating theory and practice of cross-disciplinary HPSR may inform the systematic development of new research projects and the structured assessment of existing ones. Third, a better understanding of the challenges of cross-disciplinary HPSR and potential response mechanisms may help researchers to avoid these problems in the future.

## Background

Since the early 2000s, there have been growing calls for the application of cross-disciplinary approaches to health policy and systems research (HPSR) from both governance and research circles [1, 2]. Policy-makers, health managers and researchers have consistently advocated that the scale and complexity of the twenty-first century’s health problems require disciplinary and institutional crossing [3–7]. For instance, WHO’s 2017 world report on HPSR emphasised the need for a paradigm shift towards cross-disciplinary approaches to research in the field [8]. However, research drawing on multiple disciplines is often discussed under different labels, including team science, cross-disciplinary research (CDR), multidisciplinary research (MDR), interdisciplinary research (IDR) and transdisciplinary research (TDR) [2, 9–11]. Different – and sometimes conflicting – definitions of CDR, MDR, IDR and TDR are used in the field of HPSR [6, 7, 12], which contributes to confusion about the concept and potentially has negative consequences for its further development. For instance, Kessel et al. [13] have sought to define the approach in both broad and narrow terms, first as integration of multiple disciplinary perspectives from the formulation of research questions to communication of findings, and also more broadly as the concurrent use of multiple methods.

The aim of multiple disciplinary research, subsequently referred to as CDR, is to draw together relevant conceptual, theoretical and methodological frameworks from various disciplines to enable scientific assessments of complex social problems that are more comprehensive and more relevant than what monodisciplinary approaches would achieve [14–16]. However, there are contesting and overlapping perspectives regarding the nature of cross-disciplinary integration that characterises MDR, IDR and TDR in HPSR [6, 12, 15, 17, 18]. Some scholars argue that ongoing attempts to draw distinctions among MDR, IDR and TDR are, in fact, not useful because of large overlaps across the three concepts [19–21]. However, others believe that the distinction is needed to constructively highlight differences in the degrees of cross-disciplinary integration [6, 12, 13, 22]. In the broader team science literature, reports of diverse and complex practical challenges [9, 10] call for critical and contextual synthesis to guide scholars in the HPSR field.

Without a shared understanding of the characteristics of cross-disciplinarity in HPSR, there is a risk that traditional disciplinary approaches simply persist in disguise. In addition, how to evaluate whether a given study or publication is, in fact, based on CDR has not been thoroughly investigated. These ambiguities within the literature can lead to conceptual misapplication and confusion in theory development, hence inhibiting the fundamental aims of CDR in the field. Besides these fundamental conceptual ambiguities, there are a multitude of practical challenges of cross-disciplinary HPSR [5, 7, 23–25] that do not seem easily addressable without enhanced construct clarity.

As the literature on the characteristic features of cross-disciplinarity in HPSR remains mixed, fragmented and conflicting, it provides limited guidance on why and how the approach works or does not work when applied in different contexts through different mechanisms. We believe that part of the problem is complexity. First, in health policy and systems settings, social problems are becoming increasingly complex and multifactorial just like the interventions deployed to address them [26–28]. Second, the nature and application of CDR itself is increasingly complex and contextual [2, 9, 29, 30]. Recent efforts at developing cross-disciplinary science both under the broader banner of team science [9, 31] and in health-related research [2, 29] have explored different contexts, mechanisms and outcomes of the approach, albeit without making this explicit. In practice, these components seem more integrated than isolated, thus calling for critical analysis and deeper understanding of the links between contexts, mechanisms and outcomes in CDR. This motivates a critical realist question: what is the nature of context–mechanism–outcome (CMO) configurations in cross-disciplinary HPSR and how can we assess these configurations as elements of cross-disciplinarity?

In this paper, we draw on the experiences and accounts of researchers to develop a critical synthesis of the configurations linking contexts and mechanisms with outcomes in cross-disciplinary HPSR. We aimed to critically explore the contexts in which different mechanisms of cross-disciplinarity may produce certain research outcomes. We also synthesised practical lessons on the contexts of emerging challenges and response mechanisms in this area. More specifically, the review aimed at (1) defining the characteristic features of CMO configurations in cross-disciplinary HPSR; (2) developing a framework for evaluating cross-disciplinarity in HPSR; and (3) synthesising emerging challenges of the approach that shape its contexts, mechanisms and outcomes.

## Methods

In this review, CDR is used as a broad summary term that comprises MDR, IDR and TDR (see Box 1 for operational definitions of CDR, MDR, IDR and TDR used for the review). We also used Gilson’s concept of HPSR as “*research on the policies, organizations, programmes and people that make up health systems, as well as how the interactions amongst these elements, and the broader influences over decision- making practices within the health system, influence system performance*” [1]*.* From these broad conceptual perspectives, we aimed to cover a multiplicity of worldviews as widely as possible. We used these definitions for the purpose of providing initial/basic understanding of the differences between the different notions of CDR. Thus, we did not discard any identified publication with a potentially different notion.

### Scoping the literature

An initial scoping review was conducted to obtain a general overview of the nature and scope of the literature on cross-disciplinary HPSR and to further reflect on our research aims as well as to identify and specify the range of terms for the final search. Google Scholar was the main search source for the scoping review, using two broadly conceived terms: [“Cross-disciplinary research”] AND [“Health policy and systems research”]. Following this initial review, we revised our review aims to focus on CMO configurations in cross-disciplinary HPSR and the contexts of emerging challenges and coping mechanisms.

### Critical realist synthesis

During the design of the review, we had considered using narrative synthesis. However, based on our scoping review, we identified that the review aims could not be achieved using narrative synthesis and traditional forms of systematic reviews [36–39]. We found the literature to be potentially complex and overlapping. We then explored various candidate review approaches, including scoping review [40], qualitative systematic review [41], realist review [42] and interpretive synthesis [43]. Based on discussions among the review team, we agreed to use critical realist synthesis, drawing upon a combination of realist and critical interpretive syntheses principles. While traditional systematic review methods mostly aim to produce aggregative rather than interpretive syntheses [43, 44], in this review, we aimed to closely explore the experiences and views of health policy and systems researchers on the CMO configuration, and the contexts of the emerging challenges and accompanying coping mechanisms underlying cross-disciplinary investigations in the field.

Critical realism incorporates insights from the interpretivist approach of causal explanation of interfaces among components in theory construction [45–47]. Central to our review methodology, therefore, is our (inductive) critical interpretive analysis of the interactions between the context, mechanism and outcome components of cross-disciplinary HPSR. In doing so, we draw on ideas from traditional realists reviews [42, 48, 49] and critical interpretive synthesis [43] to explore what the CMO configuration in cross-disciplinary HPSR is and how to explain the configuration. While a traditional realist review approach would focus attention on the structural dimensions of the CMO configuration in cross-disciplinary HPSR, thus potentially limiting the discourse to what the configuration is, in this review, the critical perspective to the synthesis is used to highlight how to explain the configuration. The basic structure of our synthesis is the development of heuristics for analysing and understanding the interplay between contexts, mechanisms and outcomes of cross-disciplinarity, the contexts of emerging challenges and the coping mechanisms at work.

Both realist and interpretive syntheses focus on identifying theories, which can provide guidance to the available literature [37, 43]. In realist syntheses, researchers aim to identify, within the included data, configurations of contexts, mechanisms and outcomes such that it is possible to theorise how these configurations may produce particular outcomes [37, 49]. Although realist review methodology has largely been applied in evaluating programme interventions, its focus on CMO relationships can be useful also for explaining examples of success, failure and the various eventualities in between any social interaction [37, 42, 50]. In this review, we draw on the ‘realist’ approach as it helps to focus attention on the contexts, mechanisms and outcomes that define CDR and contribute to its success or failure. Similarly, a primary principle of interpretive synthesis is that it focuses on generating theory grounded in the selected studies and not in aggregating data [43]. Thus, the interpretive dimension of this review resides particularly in our induction and critical interpretation of the internally interrelated and diverse narratives from researchers’ experiences of CDR.

### Study search and selection

Guided by the scoping review, the final list of search terms included: [“Cross disciplin” OR “Cross-disciplin” OR “Team science” OR “Multidisciplin” OR “Multi-disciplin” OR “Interdisciplin” OR “Inter-disciplin” OR “Transdisciplin” OR “Trans-disciplin”] AND [“Medical research” OR “Clinical research” OR “Public Health Research” OR “Health policy and systems research” OR “Health policy research” OR “Health system research” OR “Health policy and systems analysis” OR “Health policy and systems evaluation” OR “Health policy and systems assessment” OR “Health services research” OR “Health sciences research”].

Two reviewers first independently searched two cross-disciplinary databases – International Bibliography of the Social Sciences and Web of Science Platform – between June 20 and July 22, 2018. A date range of January 1998 to June 2018 was set. Three reviewers conducted additional searches in January 2020 in the International Bibliography of the Social Sciences, Web of Science Platform and three health-focused databases, namely PubMed Central, Embase or CINHAL. In this second search, we set the date range of January 1998 to January 2020. Our search targeted publications that were peer-reviewed original, review or commentary articles as well as institutional or government reports published in English. All forms of study designs were considered. Eligible citations were exported into EndNote X7 reference management software for screening. After removal of duplicates, the remaining citations were further screened according to the criteria set out below.

**Fig. 1:**
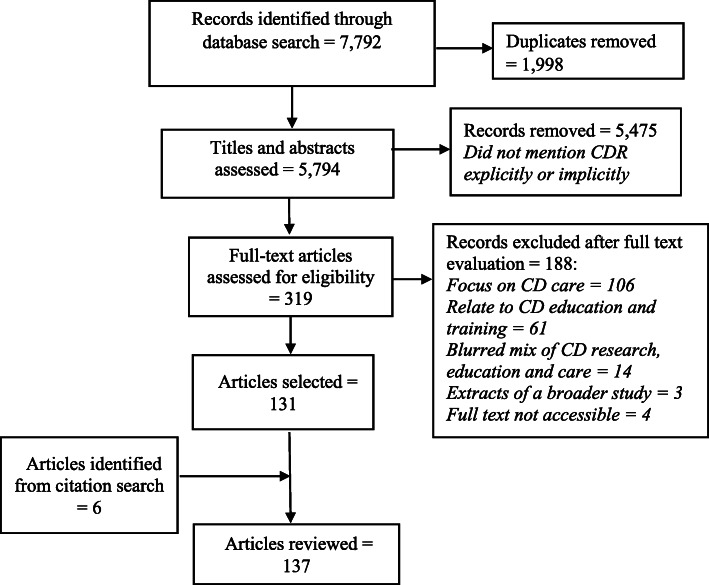
PRISMA flow diagram of literature search outcome and selection process

In step one, in view of the large number of materials remaining after removal of duplicates (Fig. 1), three reviewers independently checked the titles and abstracts to select publications whose titles and/or abstracts either explicitly described themselves as being cross-disciplinary in approach or those that could be inferred as being cross-disciplinary based on the ways in which the authors described the aims of their articles. In the second phase, the reviewers further screened abstracts of the remaining materials to exclude those that did not focus explicitly on HPSR (for instance, focused on cross-disciplinary education, training, clinical practice or care provision) or combined CDR, education and care but did not clarify findings peculiar to the research domain. We also excluded publications that were extracts of broader studies, where the report of the original investigation was accessible for review. In step three, full texts of the remaining materials were further reviewed according to the criteria set out in step two above. Eligible titles/abstracts whose full texts were not accessible were also excluded. The reference lists of studies that eventually qualified for inclusion in the final review were also searched to ensure eligible articles were not overlooked.

### Quality appraisal

As set out in the inclusion/exclusion criteria above, the selection of studies was based on their potential to adequately address the research aims. In line with Pawson’s appraisal criterion, the quality check was based on two questions. First, ‘Does the research address the theory under test?’ [48] and second, ‘Is this study good enough to provide some evidence that will contribute to the synthesis?’ [49]. A summary of the characteristics of selected studies is available for verification of the reviewers’ assessment of studies with regard to meeting these minimum quality standards (Additional file 1). All titles included in the final selection met the abovementioned criteria.

### Data extraction and synthesis

In view of the study’s aims, a three-step qualitative synthesis was adopted, inspired by Turner’s methodology of evidence synthesis [51], consisting of (1) extracting useful data on concepts and propositions; (2) synthesising points of convergence and divergence across data; and (3) making theoretical deductions from synthesis. In step one, three reviewers independently extracted data that were useful and plausible for the review. In step two, all four reviewers held a series of discussions (face-to-face, via telephone, email and Skype) to deliberate about the collected material and to organise the extracted data into main themes and categories of sub-themes, particularly highlighting areas of convergence and divergence. Where there were disagreements or conflicting themes, we reconciled them by reviewing the original texts jointly, discussing them further, and revising them as the analysis went on.

In the final step of synthesis, we drew on the emergent themes to develop theoretical insights on two aspects of the cross-disciplinary HPSR literature. The first aspect is based on the theme that the diverse concepts of cross-disciplinary HPSR reflect a CMO configuration, a dimension that has not been critically explored in the literature. In the second instance, we go beyond populating the challenges of cross-disciplinarity, as the present literature does, to illustrate the contexts and various mechanisms for responding to diverse challenges of the approach. In doing so, we aim to enable researchers to develop pragmatic coping mechanisms for addressing challenges that are likely to emerge in cross-disciplinary collaborations. We continued reviewing selected articles until we reached a point of theoretical saturation, where no new data were identified to complement the developed theory.

Box 1 Concepts of cross-disciplinary research *Cross-disciplinary research:* a general term for research that involves two or more disciplines to address a topic/problem that is beyond the scope of a single discipline [2, 32, 33].

*Multidisciplinary research:* different disciplines working discretely on different aspects of a problem to achieve a broadly gauged analysis [17, 33, 34].

*Interdisciplinary research:* robust integration of concepts, methods and perspectives from two or more disciplines to address a problem [17, 34].

*Transdisciplinary research:* a much more integrative approach involving team members from different disciplines working together and using shared concepts, methods and theories that transcend their respective disciplinary perspectives [6, 35].

## Results

### Characteristics of the selected articles

A total of 7792 titles were identified in the online search. After removing duplicates, 5794 records were screened based on titles and abstracts and 5475 records that did not explicitly or implicitly mention any of the CDR terminologies used in our search were further removed. After conducting full-text assessment of the remaining 319 materials, 188 were excluded for various reasons detailed in Fig. 1. In total, 131 articles were selected for review in addition to 6 articles retrieved from citation search. A flow chart of the entire screening and selection process is illustrated in Fig. 1.

Across the selected studies, CDR is also regularly termed as team science [52], collaborative research [53–55], multi-professional research [56] or cross-cultural health research [57, 58]. While many of the selected studies were global in scope, giving no description of the exact setting that they related to, the dominant national settings were the United States of America, Canada and the United Kingdom. The methods used in the majority of the selected studies included authors’ experiences in CDR projects, empirical studies based on completed or ongoing pilot projects, expert opinions, narrative reviews and document analyses. The studies also covered various health policy and systems issues, including mainly public health, eHealth, women’s health, maternal and child health, adolescent health, cancer care, technology and cancer health disparities, policy and care issues on aging, HIV and AIDS, and antimicrobial resistance.

The included publications generally focused on three main aspects of cross-disciplinarity, namely building understanding of the different cross-disciplinary approaches to HPSR, generating interest in the use of cross-disciplinary approaches to HPSR, and opportunities and constraints that characterize cross-disciplinarity. Generally, all studies selected for review discussed the potentials and emerging challenges of CDR in health policy and systems fields. All the selected studies also discussed some distinguishing features of cross-disciplinary HPSR. One study [59] examined the ethical considerations in cross-disciplinary HPSR and another [60] focused on knowledge brokering in cross-disciplinary HPSR. These last two areas are largely neglected in the literature. A detailed overview of the selected studies is provided in Additional file 1.

### CMO configuration in cross-disciplinary HPSR

In general, the selected studies highlight that cross-disciplinary HPSR involves the integration of multiple disciplines and methods and is characterised by a uniquely collaborative sociocultural and institutional environment. Within the selected publications, we found that cross-institutional collaboration is an (emerging) essential aspect of cross-disciplinarity. Many studies broadly consider cross-disciplinary collaborations to include both disciplinary and institutional crossing [26, 58, 61, 62], highlighting how different disciplines across multiple institutions (horizontal and vertical) can collaborate to effectively establish and address the determinants of health. In addition, the reviewed literature agrees that CDR should contribute to a better understanding of and solutions to complex health problems than monodisciplinary approaches. For some scholars, a characteristic feature of cross-disciplinary HPSR is that all relevant stakeholders participate in the study [21, 63]. However, other authors suggest that, in fact, cross-disciplinary HPSR is – or should be – defined more by the issues and questions addressed than by the particular disciplinary base or set of methods applied [22, 55, 57].

**Fig. 2:**
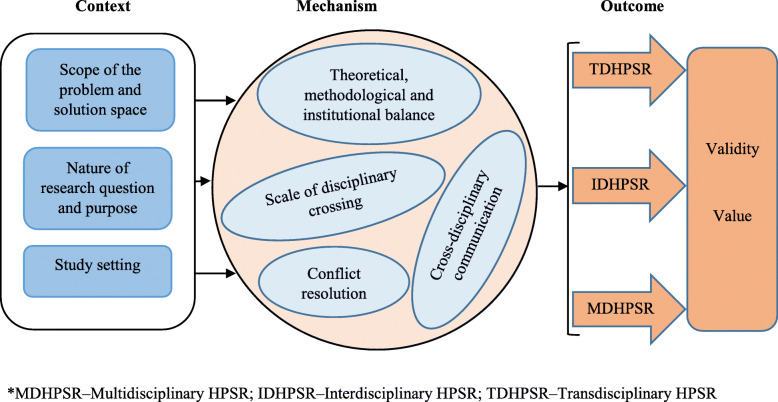
Framework of the context-mechanism-outcome onfiguration in cross-disciplinary health policy and systems research

However, taken in isolation, none of these features clearly distinguishes cross-disciplinary from monodisciplinary approaches. For instance, a mixed methodology is largely used in monodisciplinary research too. Participation of multiple stakeholders is also common in monodisciplinary studies. Our synthesis shows that the most distinguishing characteristic feature of cross-disciplinary HPSR is the configuration of particular contexts, and specific ways of working together (mechanism) to result in certain outputs of cross-disciplinarity (outcome). As illustrated in Fig. 2, the literature suggests that the outcome of a given CDR project depends on the interaction between what initiates the project (context) and how researchers design and implement cross-disciplinarity.

As presented in Fig. 2, nine themes emerged from the literature as defining characteristic features of the CMO configuration in cross-disciplinary HPSR: (context) scope of the problem and solution space, nature of research question and purpose, and study setting; (mechanism) theoretical, methodological and institutional balance, scale of disciplinary crossing, cross-disciplinary communication, and conflict resolution; and (outcome) validity and value/yield of the study. We developed the nine themes inductively from the views and narratives of authors of the studies selected for review. Overall, the literature identified cross-disciplinarity as a distinctive approach whose context, including the scope of the research problem [20–22, 24, 52, 54, 64, 65], purpose of investigation [13, 14, 23, 53, 55, 56, 66–69] and study setting [13, 24, 55, 60, 66, 67, 70–72] differ from monodisciplinary approaches. We found that these context-dependent characteristics proceed to define the mechanisms and outcomes of cross-disciplinarity [13, 20–23, 52–57, 59, 60, 64, 67, 70, 73, 74]. Our synthesis further revealed that this configuration also defines differences in the degree of disciplinary integration in HPSR and allows the development of criteria for evaluating cross-disciplinarity (see below).

### Differences in degrees of cross-disciplinary integration in HPSR

There is strong controversy in the literature about whether MDR, IDR and TDR are distinct forms of cross-disciplinary HPSR. While some scholars use the three terms/forms interchangeably or argue against their distinction [19–21], many are in favour of distinguishing between them [13, 22, 54, 57]. Interestingly, both categories of studies suggest that cross-disciplinary HPSR is generally characterised by different degrees of integration. Therefore, it is possible to use the concepts of MDR, IDR and TDR to highlight characteristic features of low versus high degrees of cross-disciplinary integration without taking a position in favour or against the distinction among them [54, 57]. From the literature, each construct may be more or less appropriate for specific contexts (such as type of research question), patterns of practice and expected research outcomes. Table 1 presents a summary of how the CMO configuration varies across the various concepts of cross-disciplinary HPSR.

**Table 1:** Different degrees of cross-disciplinary integration in health policy and systems research

Domain	Low	Cross-disciplinarity Medium	High
	MDHPSR	IDHPSR	TDHPSR
***Context***	•Applicable to health problems beyond the scope of traditional approaches [13, 19, 20, 23, 57, 70] •Disparate and discipline-specific research questions and goals [19, 20, 57] •Conceived in silos but towards a common problem [13, 23, 57, 70, 75]	•Applicable to health problems beyond the scope of traditional approaches [19, 22, 55–57, 64, 69, 70, 74] •Shared/mutually agreed-upon research questions and goals with interdisciplinary representation [19, 22, 57, 69] •Conceived based on synergy of perspectives from different disciplines [22, 29, 55–57, 64, 70, 74, 75]	•Applicable to health problems beyond the scope of traditional approaches [20, 59, 67, 70] • Shared/mutually agreed-upon research questions and goals that transcend disciplinary bases [20, 59, 67] •Conceived based on continuous learning and actionable insights beyond disciplinary boundaries [20, 67, 70]
***Mechanism***	•Integration means combining findings of individual studies conducted from different disciplinary insights [13, 21, 23, 70, 75] •Disparate intra-disciplinary investigators or teams working from their specific disciplinary perspectives [20, 21, 23] •Separate/parallel intra-disciplinary theoretical and methodological frameworks [20, 23, 73]	•Integration means collaboration between disciplines [22, 55, 56, 64, 73, 76] •Relatively basic scientific team [22, 56, 73] •Co-design, co-investigation and co-creation limited to participating disciplines [22, 57, 74] •Philosophy of transient and intermittent conceptual, theoretical and methodological integration [55, 75] •Focuses on explicit exchange of perspectives, concepts and methods [68, 74], reciprocal discipline-specific action [54, 60]	•Integration traverse stakeholder, disciplinary, organisational and professional boundaries [20, 53, 59, 67] •Formative scientific team [20, 52, 77] •Co-design, co-investigation and co-creation over disciplinary limits [20, 52, 67, 74] •Philosophy of flexibility and ongoing integration in response to new information about the problem [20, 28, 74] •Reflects robust systematic interplay between research stakeholders (academic and non-academic) and elements (design, data collection and analysis) of the research [20, 76, 78]
***Outcome***	•More basic than action-oriented output [13, 75, 79] •Output is the sum of individual evaluations [21, 23, 70] •Diverse perspectives to the topic being studied [13, 21, 23, 54]	•More comprehensive outcomes than individual parts [22, 55–57, 64, 69] •Middle-range output effectiveness and impact on problem [52, 67, 73, 75]	•Grand-scale outcome, more comprehensive and impactful [52, 67, 71, 73] •User-centred, action-oriented output [76, 77, 80] •Co-created, wholly shared reality and meaning [67, 80] •Creation of new integrated discipline [20, 67, 78]

*IDHPSR* interdisciplinary health policy and systems research, *MDHPSR* multidisciplinary health policy and systems research, *TDHPSR* transdisciplinary health policy and systems research

The literature agrees that problems addressed by all forms (notions) of CDR should be beyond the scope of monodisciplinary approaches. However, depending on the degree of cross-disciplinary integration, the research questions may differ across the involved disciplines (multidisciplinary health policy and systems research (MDHPSR)), may be shared between disciplines or have been mutually agreed upon (interdisciplinary health policy and systems research (IDHPSR) and transdisciplinary health policy and systems research (TDHPSR)). Some of the articles argue that combinations of findings from different monodisciplinary studies about a common health problem, which were not conducted in a predetermined coordinated manner, are enough to provide cross-disciplinary understanding and solutions [13, 19, 21, 23, 70]. Other studies suggest that a more conscious interactive integration of two or more disciplinary perspectives in the design and conduct of the study is needed to produce CDR outcomes [22, 55–57, 64, 69, 73, 76]. Yet, a third category of the selected studies proposes that CDR approaches should involve an iterative (continuous and repeated) integration of multiple disciplines, sectors and stakeholders to obtain an understanding of and solutions to persistent and complex systemic and policy problems [20, 30, 52, 53, 59, 67, 71, 73].

Based on the different concepts discussed in the literature, cross-disciplinary HPSR can be characterised as a continuum, ranging from MDR, to IDR and to TDR. The continuum reflects three degrees of cross-disciplinary integration, that is, additivity (MDHPSR), interactivity (IDHPSR) and iterativity (TDHPSR). In MDHPSR, cross-disciplinary output is obtained by summarizing (adding) the findings of individual discipline-specific studies, each providing a monodisciplinary viewpoint on the problem in question [13, 20, 21, 23, 70, 73]. In IDHPSR, disciplinary collaboration is based on intermittent dialogue and interactive learning in the research process [22, 54–57, 60, 64, 70, 73, 74]. Finally, TDHPSR arguably achieves the highest degree of cross-disciplinary integration by allowing for continuous and repeated (iterative) integration of new information from theory and practice such that the results of the research have a clear linkage with practice [20, 52, 53, 59, 67, 70, 74]. TDHPSR emphasises mutuality and ongoing beyond boundary reflexivity in co-design, co-evaluation and co-creation of knowledge and solutions [20, 53, 59, 67].

### Criteria for evaluating cross-disciplinarity in HPSR

Based on our CMO framework (Fig. 2) and a broad range of variables themed from the selected literature, we conceptualised a list of criteria for evaluating cross-disciplinarity in HPSR. We found that discussions around evaluation of cross-disciplinarity centred on the context, mechanism and outcome domains, which can be broken down along the nine main characteristic features included in Fig. 2. Using the nine main themes as appraisal criteria, we further inductively themed specific indicators (sub-themes) for evaluating each main theme (criterion). Overall, 28 specific indicators emerged from the synthesis. To further enhance understanding and use of our proposed criteria, we held discussions to develop illustrative evaluation questions in line with the emergent specific indicators as the review progressed. Table 3 summarises our criteria for evaluating cross-disciplinarity in health policy and systems studies.

‘Context’ generally defines the suitability or appropriateness of a CDR approach to the question or problem at hand. Cross-disciplinarity is not necessarily applicable in every HPSR project. Therefore, in evaluating cross-disciplinarity, it is important to establish whether (1) the nature of the problem and expected outcomes, (2) the accompanying research question and purpose of investigation, and (3) the setting are beyond the scope of monodisciplinary approaches. We found eight specific indicators for evaluating whether these conditions are fulfilled in a given study (Table 2). The indicators include the justification of a cross-disciplinary approach to address the problem, the fit of research question with a cross-disciplinary perspective, and the justification of the need for a cross-disciplinary study in the specific setting.

**Table 2:** Criteria for evaluating cross-disciplinarity in health policy and systems research

Domains	Appraisal criteria	Specific indicators and references	Illustrative evaluation questions
***Context***	Scope of the problem and solution space1.	Articulation of the problem [13, 22, 24, 53–55, 71, 73, 92]	Has the problem been adequately described, in terms of its nature, scope and relevance?
		Justification of a cross-disciplinary approach to the problem [23, 54, 55, 69, 70, 76]	Is there a clear description of how cross-disciplinarity provides a more useful approach to addressing the problem than the alternatives?
	Nature of research question and purpose1.1.1.	Fit of the research question with cross-disciplinary perspective [53, 54, 67, 73, 85]	Does the frame of the research question reflect a cross-disciplinary perspective?
		Statement of expected cross-disciplinary outcomes [26, 52, 57, 62, 93, 97]	Are the expected cross-disciplinary outcomes of the research explicitly stated?
		Appropriateness of the study purpose for addressing the research question [53–56, 67, 73, 85]	To what extent does the research purpose reflect the cross-disciplinary question under investigation?
		Conceptualisation of research question and purpose [28, 53–57, 63]	Does the analysis and/or frame of the research question and purpose demonstrate critical consideration of theory and evidence from multiple disciplines relevant to the context?
	Study setting1.	Description of study setting [29, 53, 57, 68, 70, 71]	Does the study setting as described reflect the research problem and question specified?
		Justification of setting for cross-disciplinary cross-disciplinary study [53, 55, 60, 67, 70]	To what extent does the context of the setting necessitate a cross-disciplinary approach?
***Mechanism***	Theoretical, methodological and institutional balance 1.1.1.1.1.1.	Representation of multiple disciplinary perspectives [13, 19, 20, 23, 52, 53, 55, 56, 63, 66, 68, 69, 71–73]	Have the diverse disciplinary orientations been clearly defined to allow for evaluation?
		Balance of institutional representation [52, 54, 56, 66, 69, 70, 76]	If the research crosses institutional boundaries, are the relevant institutions and disciplines clearly stated in the study?
		Means of integrating diverse perspectives, theories and methods [28, 53, 56, 59, 67, 73]	Are the methods for disciplinary integration clearly defined?
		Fit of methods for purpose [26, 29, 58, 77, 92]	Do the methods for generating and analysing data fit a cross-disciplinary strategy?
		Criteria for selection and composition of the scientific team [19, 20, 54, 57, 60, 69, 73]	Are the criteria used for selecting the team suitable for the research problem?
		Fit of team members’ backgrounds to the research problem and solution space [13, 20, 21, 23, 52, 57, 63, 67, 68, 72]	Do the team members’ background and expertise fit the problem space?
		Appropriate distribution of responsibility [19, 24, 54, 56, 69, 70]	Does the definition of participating stakeholders’ roles reflect their disciplinary representation in the project?
	Scale of disciplinary crossing1.	Extent of cross-disciplinary knowledge integration [13, 19, 22, 52, 67, 68, 70, 71, 76]	To what extent (high/medium/low) does the study cross diverse disciplines or fields?
		Stages of disciplinary crossing in the research process [52, 53, 56, 67, 72]	At what stage of the study are disciplinary boundaries crossed?
	Shared (cross-disciplinary) communication 1.1.	Commitment to a cross-disciplinary communication framework [13, 21, 56, 57, 63, 68, 70, 72, 79]	Are there clear efforts to ensure effective cross-disciplinary communication throughout the project?
		Indication of collegial decision-making among team members [19, 30, 53, 56, 62]	Does the design have mechanisms in place to allow for collective decision-making?
		Interactive and iterative process [52, 53, 59, 69, 71, 76, 79]	Does the communication structure reveal clear potential for team members to regularly interact and jointly evaluate their assumptions and processes?
	Conflict resolution1.	Identification and response to researchers’ own conflicts [19, 23, 24, 58, 59, 67, 68, 70]	Are the possible/practical disciplinary and personal conflicts among researchers stated and addressed?
		Explicit indication of cross-disciplinary/institutional ethics [24, 54, 58, 59, 62, 70]	Have the different disciplinary and institutional ethical positions and assumptions been adequately explained and addressed?
***Outcome***	Validity 1.1.	Achievement of cross-disciplinary purpose [52, 55, 69, 76, 98]	Has the cross-disciplinary purpose of the research been fulfilled?
		Cross-disciplinary balance was maintained as planned [27, 58, 77, 80, 91, 95]	Has the intended disciplinary and institutional balance been adequately adhered to?
		Consistency/coherence of common vocabulary [19–22, 24, 52, 54, 57, 67, 71, 72]	Do the research results convincingly represent an interplay of the different disciplinary voices?
	Value/Yield	Additional contribution (value addition) [13, 21, 26, 52–54, 56, 67, 69, 92, 98]	Has extra scientific and social impact been demonstrated beyond that attainable through monodisciplinary approaches?
		Problem-solving capacity [21, 30, 54, 57, 63, 64, 66, 73]	Do the study results contribute to a fundamental understanding of the problem or actionable solutions or a combination of both?
		Novelty or innovation [55, 64, 67, 68, 71, 74, 79]	Have the research results demonstrated novelty by virtue of the use of a cross-disciplinary approach?

Analysis of the ‘mechanism’ element of cross-disciplinarity is based on four appraisal criteria, which focus on the structures and processes of disciplinary integration. First, seven indicators define theoretical, methodological and institutional balance. Second, two indicators can be used to assess the scale of disciplinary crossing, including the degree of cross-disciplinary knowledge integration and the stages of disciplinary crossing. Third, we found three indicators for assessing whether researchers have an approach for shared (cross-disciplinary) communication, all highlighting commitment to a common language throughout the project, such as whether they have developed common vocabulary, and refined their terminology through an iterative process. Finally, a fourth appraisal criterion is about the process for addressing potential conflicts, which can be evaluated using two indicators – first, whether and how the researchers have explained and addressed their own dilemmas and biases, and second, an explicit indication of cross-disciplinary/institutional ethics.

The review further identified two appraisal criteria and six indicators for assessing the ‘outcome’ of cross-disciplinarity in HPSR. The first three indicators assess the validity of the study’s cross-disciplinarity claims by focussing on whether (1) the cross-disciplinary purpose was achieved, (2) the planned cross-disciplinary balance was maintained, and (3) a common vocabulary was adhered to. The remaining three indicators focus on establishing that the study produces additional scientific value and novel results in addressing the targeted health problem compared to what monodisciplinary approaches could have achieved.

### Challenges of cross-disciplinary HPSR

Generally, all the articles reviewed highlighted several challenges of cross-disciplinary HPSR, which can be classified under five main inter-related themes, namely conceptual challenges, institutional challenges, challenges related to communication and information management, coordination challenges, and resource challenges. However, the literature reveals numerous interactions among these challenges, which were often multifactorial and required delicate negotiations to be overcome. In this section, a description is given of the practical mechanisms for responding to these challenges (illustrated in Table 3).

**Table 3:** Emerging challenges of cross-disciplinary health policy and systems research

Challenges	Examples	Coping mechanisms
***Conceptual***	Difficulty in developing a shared conceptual framework [29, 30, 56, 67, 68, 70, 80, 81]	Establishing a CDR learning platform [30, 53, 67, 70, 76, 82, 83] Developing a well thought-out rationale for cross-disciplinary research [54, 55, 57, 60, 67] Mentoring for early career researchers [27, 53, 54, 70, 76]
	Disagreement about research methods [56, 57, 61, 67, 68, 80, 82]	Piloting multiple methods [53, 57, 68, 76, 84] Seeking inputs/counsel from (experienced) external advisors [61, 81, 85, 86]
	Challenges in maintaining disciplinary balance as the study progresses [56, 57, 66, 67, 70, 81, 87]	Sustaining interaction, dialogue and ongoing evaluation of the process [60, 66, 67, 70, 76, 82] Pre-developing mechanism for cross-disciplinary revision [53, 54, 57, 83, 88]
	Disciplinary capture; politics of hierarchy of disciplines [24, 56, 70, 86]	Building shared understanding of participating disciplines [30, 56, 73, 82, 86] Modifying established research practices to fit multiple disciplinary contexts [26, 27, 83, 86, 89]
***Institutional***	Career progression and promotion criteria remain discipline based [52, 56, 59, 68, 69, 79]	Building multi-stakeholder support for cross-disciplinarity [19, 54, 55, 67, 69, 79]
	Suiting financial requirements of funding bodies [24, 54, 55, 64, 66]	Developing internally generated funding avenues [55, 60, 88] Diversifying funding networks [24, 60, 64, 66, 88]
	Reluctance among institutions to cross-disciplinarise [53, 59, 60, 70, 90]	Building a cross-disciplinary organisational culture in the overall health policy and systems structure [29, 53, 60, 62, 70, 76, 79] Reaching out to government, industry and community [52, 56, 60, 67, 76, 80, 88]
	Limited external stakeholder appreciation of cross-disciplinary research potentials [53, 57, 60, 73, 81, 91]	Demonstrating the extra scientific impact of cross-disciplinary research over intradisciplinary research [53, 55, 70, 73, 81] Maintaining knowledge brokering with external stakeholders [60, 74, 80, 81, 88, 91]
***Communication and information management***	Frequent communication breakdowns and disenchantment among team members [19, 23, 52, 66, 73, 81]	Keeping to a shared cross-disciplinary communication plan from the onset [23, 57, 60, 73, 92] Ensuring frequent interaction and meetings among team members [23, 60, 73, 81, 85] Using interactive media to develop team communication platforms [23, 60, 73, 79]
	Challenge of accommodating discipline-specific languages of participating disciplines and stakeholders [19, 56, 59, 67, 93]	Focusing more on development of a shared language than capturing each discipline’s specific tone [19, 26, 67, 85, 87]
	Challenge of finding media suitable to participating disciplines and stakeholders [19, 56, 59, 67]	Using various communication mediums and strategies [19, 26, 81]
	Conflicts over authorship and publication of research output [19, 58, 59, 67, 68]	Formulating authorship guidelines from the onset of the project [19, 56, 58, 76, 79, 85] Breaking down overall team into smaller similar-interest teams [52, 59, 67, 76, 87]
***Research coordination***	Difficulties in avoiding and addressing interpersonal conflicts [56, 58, 60, 67, 69, 70]	Developing strong and trusted team leadership [53, 54, 76, 87, 94] Fostering interdisciplinary relations [54, 56, 58, 67, 69, 70] Maintaining manageable team sizes [24, 60, 70] Facilitating small group/problem-solving sessions [57, 60, 67]
	Difficulty in obtaining commitment and participation from team members [24, 68, 70, 76, 95]	Blending formal and informal structures to boost morale [24, 53, 76] Negotiating and building compromises [55, 57, 73, 76, 82, 87] Clearly articulating team members’ roles early in the process [53, 54, 57, 88] Developing an evaluation criterion from the outset [23, 63, 66, 70, 73]
	Marginalisation and power dynamics [56, 69, 76, 81]	Building trust on fair disciplinary representation [30, 67, 68, 81, 95] Defining authority–responsibility parity early [54, 56]
	Challenge of integrating diverse and incompatible ethical codes [58, 59, 67, 92, 95, 96]	Promoting mutual knowledge and respect for intradisciplinary ethical codes [58, 59, 67, 92, 95, 96]
***Resources***	Heavy time commitment to team meetings and study schedules [24, 52, 53, 68, 82, 90]	Obtaining institutional commitment to time and workload adjustments for cross-disciplinary researchers [24, 56, 58, 76, 82, 94] Setting reasonable research goals and timelines [24, 53, 57, 67, 70]
	Balancing research commitments with work requirements [52, 53, 67, 74]	Integrating cross-disciplinary research into existing institutional structures [24, 52, 57, 61, 69]
	High cost of research [22, 23, 67, 76, 80]	Building external stakeholder support [22–24, 53, 59–61, 79, 88]

A dominant theme in the literature is the challenge of developing a shared concept of cross-disciplinary HPSR. The most commonly cited conceptual challenges were a difficulty in developing a shared research question and purpose [23, 56, 64, 65, 67, 68, 70, 81], disagreement about research methods [56, 57, 67, 68], challenges in maintaining disciplinary balance [56, 57, 66, 67, 70], and the prevalence of disciplinary capture superimposed by traditional health disciplines on contributory disciplines [23, 24, 56, 64, 65, 70]. The latter is noted to create politics of hierarchy of disciplines for CDR in the field [26, 29]. Many of the selected studies identified that developing a shared investigation framework from the onset of the study offers opportunities to effectively address conceptual barriers [53, 54, 57, 60, 66–68, 70, 76].

Institutional challenges mainly relate to prevailing structures that are not open to cross-disciplinary approaches. Securing cross-disciplinary recognition and support remains problematic [20, 65]. Institutional policies on disciplinary focus of faculty [54], authorship and intellectual property restrictions [54] and limited funding support [64, 68] are significantly undermining cross-disciplinary efforts in HPSR. Researchers also face an enormous challenge of suiting the (often stringent) funding requirements of external funding bodies [24, 64, 66]. For instance, some funding agencies demand that the research team consists of members from specified disciplines and institutions, often resulting in irrelevant artificial teams and undesirable research outcomes [54, 55]. Others set strict time and budget lines for the investigation, which are often not realistic for a truly cross-disciplinary approach [24]. In addition, many institutions remain reluctant to cross-disciplinarise [6, 14, 19, 21], coupled with limited external stakeholder appreciation of CDR potentials [3, 16, 19, 21].

Cross-disciplinary HPSR requires particular communication mechanisms (Table 2) but institutional and cultural differences often hamper effective communication throughout cross-disciplinary HPSR projects [19, 22, 23]. As the research progresses, communication dynamics may stray due to the multiplicity of stakeholder interests in health settings [19, 23, 52, 66, 73]. Knowing what and how best to convey the results and diverse opinions of the research team devoid of dominance by a single discipline is a great challenge, especially for early career researchers [99]. Using different disciplinary languages and media to disseminate research results to different target groups is another major challenge [19, 54, 56, 59, 67]. Agreeing on a publication strategy (choice of journal and order of authors) is often also a very controversial communication issue [19, 59, 67, 68, 99]. Addressing the communication challenge requires having a shared communication and information management plan from the onset of the research [23, 57, 60, 73], which may be supported by the use of non-traditional interactive media to develop shared communication platforms [23, 60, 73, 79].

In relation to coordination, the key challenges relate to bringing individual team members’ qualities, perspectives, cultures and expectations to fit with the cross-disciplinary focus. Besides unavoidable interpersonal conflicts [56, 60, 67, 69, 70], principal investigators (PIs) often have to deal with poor commitment and participation from team members [24, 68, 70, 76]. Two studies [59, 67] particularly highlighted difficulties in integrating diverse and incompatible ethical codes. Some studies [24, 69] pointed to gender differences as a coordination challenge, especially for women PIs. Good team leadership [24, 53, 54, 60, 70, 76] and clear articulation of the coordination agenda from the start of the research [23, 24, 54, 56, 63, 66, 67, 69, 70, 73] are the most commonly advocated mechanisms for dealing with coordination challenges in cross-disciplinary HPSR. In one study [53], both practitioners and researchers identified good personal traits and skills of the PI and research team as the most critical influences of effective coordination.

Resource challenges for cross-disciplinary health researchers are also important. The costs of CDR in terms of time, effort and money are higher than those of monodisciplinary research [21–23, 67, 71, 76]. For upcoming scholars, the most significant challenge to their participation in cross-disciplinary HPSR is the difficulty in accessing funding for their projects [21, 23]. Additionally, cross-disciplinary HPSR scholars have to balance their research commitments with organisational responsibilities [21, 24, 52–54, 67, 74]. The time lag between commencement and completion of cross-disciplinary HPSR projects also poses significant challenges for young researchers [24, 52, 53, 57, 63, 68]. Beyond the research teams’ management of time and effort [24, 53, 57, 67, 70], broader stakeholder engagement is needed to promote cross-disciplinary HPSR funding and buy-in [22–24, 52, 53, 56, 57, 59, 60, 69, 76, 79].

## Discussion

### Summary of key findings

Our review shows that there is growing interest in the concept and application of cross-disciplinary approaches in HPSR. The review provides a pathway for the further development of cross-disciplinarity in HPSR, and it may help practitioners, policy-makers and researchers better understand the characteristic features, evaluation criteria and emerging challenges of cross-disciplinary HPSR. In particular, the review makes three important contributions to the literature. First, it has developed a conceptual and analytical framework for understanding the CMO configuration in cross-disciplinary HPSR – a neglected aspect of cross-disciplinary evaluation in the broader team science literature [2, 9, 15, 100, 101]. Second, it has defined criteria for assessing whether a HPSR project is, in fact, cross-disciplinary. Third, the review has summarised existing challenges of cross-disciplinary HPSR and shared experiences of researchers in addressing them.

The CMO framework of cross-disciplinary HPSR draws attention to the fact that the approach is not defined by individual characteristics but by the combination of a particular type of research question and setting (context), a specific way of researchers working together (mechanism), and a certain research output (outcome) that is superior to what could be achieved under a monodisciplinary approach. Existing analysis of integration in CDR have not clearly illustrated the unique influence of CMO configurations in the integration process [15, 102, 103]. Our analysis of MDHPSR, IDHPSR and TDHPSR as three examples of cross-disciplinary approaches characterized by different CMO configurations on a continuum of increasing disciplinary integration allows a pragmatic debate about the appropriate degree of integration for a particular research problem and purpose.

While the criteria do not claim to evaluate research quality as several studies in the broader CDR literature have provided significant guidance in that regard [10, 15, 16, 34, 104], the 28 proposed indicators allow to assess whether a given research project actually demonstrates integration of disciplinary knowledge, i.e. whether it is truly cross-disciplinary. In summary, the proposed criteria aim to assess three basic questions. First, does the context of the investigation warrant a CDR approach? Second, to what extent does the study demonstrate cross-disciplinary effort, in terms of its research structures and processes? Finally, is the (intended) research outcome superior to what could be achieved through a monodisciplinary approach? Given that the appropriate degree of cross-disciplinary integration depends on the specific research question, the proposed criteria and indicators should not be understood as demanding a predefined level of integration but rather as specifying different domains for assessing the appropriate degree of integration.

In other words, drawing upon the broader team science literature [2, 6, 9, 10, 17, 35, 104], it is our position that integration of disciplinary knowledge and/or multiple stakeholder inputs is a means to serve a specific research/project purpose rather than an end in itself. Therefore, the relevance and adequacy of individual indicators depends on the specific purpose of a project under evaluation. Sensitivity to the context of integration is a fundamental principle of evaluation [105]. Consequently, we do not advocate that all 28 indicators be necessarily applied to every cross-disciplinary project.

Cross-disciplinary HPSR is widely acknowledged as a very challenging endeavour. This review shows that challenges in the field relate to conceptual, institutional, communication and information management, coordination and resources. While similar findings have been established in studies outside HPSR settings [14, 16, 17, 33, 99, 104], cross-disciplinary HPSR projects and teams often have unique contexts and face peculiar challenges [5, 6, 25] and this review offers guidance to overcoming some of these complex contextual challenges. Our synthesis of the contexts and mechanism of emerging challenges and the strategies used to mitigate them presents a useful map for achieving cross-disciplinary outcomes. This review also underscores the need for critical attention to the delicate interactions among cross-disciplinary challenges and their related coping strategies.

### Implications for policy and research

Previous attempts at characterising cross-disciplinary HPSR have overly focused on comparing different concepts of cross-disciplinarity (MDR, IDR, TDR), often claiming superiority of one over the other. Others have looked at cross-disciplinarity in the limited context of mixed-methodology. For instance, O’Cathain et al. [100] and De Allegri et al. [106] focus on mixed methods evaluation in HPSR. Our framework may contribute to overcoming competing claims for superiority of individual approaches by highlighting that the different approaches are characterised by increasing degrees of disciplinary integration, which means that they can complement each other depending on the specific research contexts.

There are many examples of evaluation frameworks in the cross-disciplinary literature. However, we find that they are overly generic [104, 105] or based on indirect indicators like authors’ grant awards and citations of the work under review [14, 97, 107]. For policy-makers, researchers and research funders, our evaluation criteria provide much more specific and clearer guidance for assessing previous, ongoing or future cross-disciplinary HPSR projects. For example, researchers may use the criteria in new research proposals to more clearly define the research context, the envisaged mechanisms of working together, and the expected outcomes of cross-disciplinary HPSR. The criteria may also help to improve ongoing research projects by assisting researchers to identify broader challenges commonly associated with cross-disciplinarity. Finally, the framework can serve as a practical tool to help policy-makers know whether research proposals/findings are based on CDR ideals or not.

The challenges and potential solutions for cross-disciplinary HPSR synthesised in this review may also provide guidance in the design and implementation of future research projects. As challenges vary across contexts, developing a context-driven research management plan may help to avoid these from the outset of new projects [53, 55, 57]. Doing so from a multilevel analytical perspective will help capture the interface among institutional, social and researcher personality challenges of cross-disciplinarity [28]. The review also highlights the need for the wider health policy and systems community, including researchers and research institutions, government, policy-makers, health organisations, health managers, funders, journal editors and all relevant stakeholders in health, to contribute to effective cross-disciplinarity. For instance, cross-disciplinary researchers can enhance multi-stakeholder understanding, interest and commitment through knowledge brokering [58, 60]. This review provides guidance on how researchers can lead the process by conducting CDR in ways that potentially meet multi-stakeholder informational needs.

Going forward, two main areas need priority attention in future research; first, developing methodological guidance on how to perform cross-disciplinary HPSR should attract priority attention to assure further advancement of the field. We acknowledge that some important efforts in that regard have emerged [2, 26]. However, much work is needed to advance understanding of diverse contexts/aspects and their configurations. This review is our contribution in that direction. Second, cross-disciplinary HPSR should develop understanding of how macro (social and institutional) and micro (individual personality) level factors/contexts interact to facilitate/hinder operationalisation of the approach. We believe that such an integrated approach is able to more critically explore the web of defining factors for cross-disciplinary success than isolated endeavours. We advocate research that emphasises multiple levels of analysis to help clarify such causal links (configurations) in order to promote more pragmatic learning on success and sustainability.

### Limitations of the review

First, despite the extensive literature search conducted, as cross-disciplinary HPSR is noted to generally be an emerging approach, our search may have excluded studies that did not use the CDR terminologies applied in our search. We also recognise that articles that are cross-disciplinary may not have (explicitly or implicitly) described themselves as such in their titles/abstracts. However, based on our scoping review, the terminologies applied in our search are the most commonly used in both classic and recent literature [2, 32]. In addition, if articles did not describe themselves as cross-disciplinary in the title/abstract it would have required full text review of the thousands of citations generated, and this presented practical limitations to the reviewers. Further, our objective in this review was to find illustrative examples of publications that exemplify the CMO configurations and challenges of cross-disciplinary HPSR rather than to explore all aspects of the literature in the field.

Second, the review was also limited to articles published in English and much of the literature is anecdotal or based on description of pilot projects, raising concerns about the generalisation of our framework. Despite these limitations, given the wide similarities across studies captured in this review, we believe that our findings adequately synthesise the most important themes in the existing literature. In addition, although our selection of the most important challenges was subjective but based on available literature, there was wide agreement across studies about emerging challenges of the approach in the HPSR field. We recognise that this review should be taken as a starting point for a broader discussion about the concept, theory and evaluation of cross-disciplinary HPSR, which may ultimately lead to the development of quality assessment and reporting guidelines similar to those offered by the EQUATOR network (see, for example, O’Cathain et al. [100] and Belcher et al. [15]).

## Conclusion

To the best of our knowledge, this review is the first to synthesise the existing literature on cross-disciplinary HPSR using a realist CMO perspective. We hope to ignite a discourse on the role of CMO configurations in influencing contested notions and operationalisations of cross-disciplinarity. In doing so, we make three important contributions to the literature by (1) conceptualising the CMO configuration of cross-disciplinary health policy and systems research; (2) developing criteria for evaluating cross-disciplinarity; and (3) synthesising potential response mechanisms to emerging challenges of the approach.

These contributions have important implications for researchers and decision-makers. First, a better understanding of the characteristic features of cross-disciplinary HPSR (including IDR, MDR and TDR) is essential for researchers to conduct high quality investigations and for policy-makers to engage with researchers in ways that engender translational research outcomes. Second, given the increasing complexity of the cross-disciplinary knowledge field, we have presented criteria for evaluating both theory and practice of cross-disciplinary HPSR. Policy-makers and researchers can use the criteria for the systematic development of new CDR projects and for a structured assessment of existing ones, ultimately contributing to better quality of future HPSR projects. Third, deeper understanding of the challenges of cross-disciplinary HPSR and potential response mechanisms may help (early career) researchers to manage similar situations, thus avoiding common deficiencies.

Further advancement of cross-disciplinary HPSR will require creativity and innovation to effectively navigate the growing complexity of cross-disciplinarity itself and the health and social problems targeted for investigation. The CMO framework of cross-disciplinary HPSR signals opportunities for developing understanding of whether/how different elements of cross-disciplinarity interact to produce greater and innovative impact on policy and practice. As Tannen notes, “*The only way we can make sense of the world is to see the connections between things*” [108].

## Supplementary information

**Additional file 1.** Summary of characteristics of studies included in the review.

## Data Availability

All data generated or analysed in this study are included in this published article and its supplementary information files.

## Abbreviations

HPSR: Health policy and systems research
CMO: context-mechanism-outcome
CDR: cross-disciplinary research
MDR: multidisciplinary research
IDR: interdisciplinary research
TDR: transdisciplinary research
IBSS: International Bibliography of the Social Sciences
WSP: Web of Science Platform
MDHPSR: multidisciplinary health policy and systems research
IDHPSR: interdisciplinary health policy and systems research
TDHPSR: transdisciplinary health policy and systems research
