# Supplementary material for: A critical realist synthesis of cross-disciplinary health policy and systems research: defining characteristic features, developing an evaluation framework and identifying challenges
Source: Health Res Policy Syst. 2020 Jul 14;18:79. doi: 10.1186/s12961-020-00556-2 (PMC7359589; doi:10.1186/s12961-020-00556-2)
Supplement: Supplementary file 1 — Additional file 1. Summary of characteristics of studies included in the review. [file 12961_2020_556_MOESM1_ESM.docx]

**Supplementary Material 1: Summary of characteristics of studies included in the review**

| **Reference** | **Setting/Country** | **Design/Method** | **Dominant CDR Concept Used** | **Focus of Study/Article** |
| --- | --- | --- | --- | --- |
| Kessel et al (2009) [1] | United Kingdom | Mixed methods study involving quantitative (geographical information systems) and qualitative (ethnographic) analysis | Multidisciplinary research (MDR) | Exploring potential of deepening understanding of multidisciplinary health research |
| Dankwa-Mullan et al (2010) [2] | Global | Narrative literature review | Transdisciplinary research (TDR) | Developing a framework for integrating cross-disciplinary approaches in health disparities research |
| Smith and Master (2017) [3] |  | Narrative literature review | Multi/interdisciplinary research | Developing a framework on distribution of contributorship and authorship in multi/interdisciplinary health research |
| Grönqvist et al (2017) [4] | Sweden | Descriptive case study | MDR | Description of challenges of a publicly funded multidisciplinary health research environment |
| Hubbard (2006) [5] | Global | Qualitative analysis based on researcher experience and literature | Interdisciplinary research (IDR) | Examination of the application of interdisciplinary health services research |
| Clarke et al (2012) [6] | United Kingdom | Grounded theory | IDR | Highlighting lessons on the process and challenges of inter-disciplinary health research |
| Mazure et al (2000) [7] | USA | An exploratory study based on literature and the researcher experience | IDR | Generating and sustaining interest in use of multidisciplinary research in women’s health |
| Canadian Academy of Health Sciences (2005) [8] | Canada | An exploratory study using literature and the Canadian experience | IDR | Promoting IDR in health sciences and enhancing understanding of the approach in Canada |
| Raghunath and Innes (2005) [9] | Global | Narrative analysis based on literature and researcher experience | MDR | Exploring the meaning, usefulness and reality of MDR in primary healthcare |
| Bindler et al 2012 [10] | USA | Exploratory case study | IDR | Exploration of the strengths, challenges and facilitative approaches of interdisciplinary research in health sciences |
| Carey and Smith (2007) [11] | Australia | Qualitative analysis based on researcher experience | IDR | Discussion of the role of interdisciplinary health researchers |
| Slatin et al (2004) [12] | USA | Exploratory study of ongoing CDR project | IDR | Mechanisms and pathways influencing occupational health disparities among healthcare workers |
| Giacomini (2004) [13] | Global | Narrative analysis based on literature and researcher experience | IDR | Highlighting sustainable ways of tackling the methodology and practice of interdisciplinarity in health research |
| Aagaard‐Hansen and Ouma (2007)[14] | Global | Narrative analysis based on literature and CDR experiences of researchers | CDR | Discussion of key practical and theoretical issues in the management of interdisciplinary health research |
| Nair et al (2008) [15] | Canada | A descriptive study using qualitative interviews | IDR | Establishing researchers’ perspective on conceptualization and Operationalization of interdisciplinary health research |
| Domino et al (2007) [16] | USA | Ethnography | IDR | Dealing with the challenges and opportunities of interdisciplinary women health research |
| Urquhart et al (2013) [17] | Global | Narrative review | CDR | Discussion of how cross-disciplinary approaches can optimize the use of research in cancer care |
| Laberge et al (2009)[18] | Canada | Mixed methods empirical analysis of practitioner support for or against CDR | IDR | Examining biomedical and clinician  scientists’ views about the value of interdisciplinary  health research |
| Pincus et al (2007)[19] | USA | Exploratory case study | IDR | Assessing initiatives to promote interdisciplinary geriatric healthcare research |
| Marts (2002)[20] | USA | Analysis of panel presentations from a Women’s Health Research Meeting | IDR | Describing the prospects and barriers to progress in research on sex-based differences in health |
| Lohfeld and Brazil (2000)[21] | Canada | An empirical exploratory mixed methods study | Collaborative research | Building understanding on elements needed to develop and maintain effective collaborative  research in healthcare for older adults |
| Gebbie et al (2008)[22] | USA | Empirical qualitative study based on Delphi-method | IDR | Identifying the competencies required for successful  interdisciplinary health research |
| Aagaard-Hansen and Ouma (2002)[23] | Kenya | Qualitative analysis based on researcher experience | IDR | Discussion of practical and theoretical issues in the management of interdisciplinary health research |
| Aagaard-Hansen et al (2004)[24] | Global | An analytical discussion of emerging ethical concerns in CDR | CDR | Examination of key ethical aspects of CD health research |
| Magnus and Castel (2016)[25] | Global | Narrative literature review | MDR | Characterising the  importance of multidisciplinary teams in HIV research |
| Magill-Evans et al (2002)[26] | Canada | An analytical discussion based on researcher experiences from a pilot TDR project | TDR | Discussion of strategies used to cope with challenges in undertaking TDR in childhood disabilities |
| Larson et al (2005)[27] | USA | Empirical qualitative study of an ongoing IDR project | IDR | Highlight of conceptual underpinnings and process for developing ID approach to antimicrobial resistance research |
| Urquhart et al (2011)[28] | Canada | Analysis based on researcher experiences in an ongoing MDR project | MDR | An in-progress perspective on knowledge brokering within a MDR team working on improving access to quality colorectal cancer care in Canada |
| Albrecht et al (2006)[29] | USA | Commentary | TDR | Developing TDR methods in treatment decision-making. |
| An et al (2007)[30] | USA | Comparative case analysis | TDR | Providing guidance on the development of transdisciplinary research in biomedical fields. |
| Fletcher et al (2017)[31] | UK | Exploratory case study based on interviews | Collaborative research | Discussion of the factors that enable or impede collaborative research |
| Cooper et al (2013)[32] | USA | Embedded case study | TDR | Developing an operational model for CDR in hypertension disparities in African American population. |
| Corbett et al (2013)[33] | USA | Descriptive case study | IDR | Discussion of barriers and facilitators to designing, conducting, and translating interdisciplinary research |
| Denne et al (2013)[34] | USA | Comparative case study design | IDR | Providing guidance on use of pilot funding to stimulate IDR |
| Goldsack et al (2016)[35] | USA | Qualitative analysis of semi structured interviews | IDR | Investigating how surgeons make decisions about engaging in CDR, and identifying underpinning facilitators and barriers |
| Grey & Connolly (2008)[36] | Global | Commentary | TDR | Describe the development of transdisciplinary clinical research in and public health. |
| Grigorovich et al (2016)[37] | Global | Narrative review | TDR | Synthesis of literature on operationalisation, barriers and enablers of TDR |
| Harper et al (2008)[38] | USA | Exploratory mixed methods design | TDR | A mechanism for evaluating the successes and challenges of transdisciplinary research |
| Harris et al (2011)[39] | Global | Citation network analysis and expert review | MDR | Examination of cross-disciplinarity public health services and systems research through a citation network approach |
| Hebert et al (2009)[40] | Global | Narrative review | IDR, and Community-Based Participatory Research (CBPR) | Deepening understanding of CBPR and interdisciplinary engagement. |
| Hills & Richards (2014)[41] | Global | Narrative review based on interviews with interdisciplinary researchers | IDR | Exemplifying processes, facilitators and impediments of IDR |
| Holmes et al (2008)[42] | USA | Descriptive case study | TDR | Challenges of TDR in health disparities investigations |
| Kessel & Rosenfield (2008)[43] | Global | Narrative review | TDR | The evolution, constraints and facilitators of transdisciplinarity in health research |
| Kestenbaum et al (2015)[44] | USA | Autoethnography | IDR | The role of chaplains in IDR on palliative care |
| Iida et al (2005)[45] | USA | Descriptive case study | MDR | Opportunities and challenges of multidisciplinary, multisite research collaborations |
| Kobus & Mermelstein (2009)[46] | USA | Descriptive case study | TDR | Developing insights on TDR policy |
| Lakhani et al (2012)[47] | Global | Literature review | IDR | Examination of the attributes effective cross-disciplinary teams in health research |
| Mabry et al (2008)[48] | USA | Descriptive case study | IDR | Offering directions on how IDR can promote public health delivery |
| Neill (1999)[49] | Global | Narrative review | IDR | Developing understanding of effective IDR process |
| O'Cathain et al (2008)[50] | Global | Exploratory study using semi structured interviews | MDR & IDR | Facilitators of and barriers to cross-disciplinary health research |
| Person et al (2018)[51] | USA | Descriptive case analysis | CDR | Designing cross-disciplinary investigation |
| Ranford et al (2019)[52] | USA | Exploratory case study | IDR | Occupational therapists’ role in a collaborative, interdisciplinary research study |
| Schultz et al (2011)[53] | USA | Descriptive case study | IDR | Building interdisciplinary research centres in geriatric healthcare |
| Tran et al (2019)[54] | Global | Bibliometric and content analysis | IDR | Global mapping and analysis of HIV/AIDS research |
| Turnbull et al (2019)[55] | Global | Literature review | TDR | Establishing best practice TDR methods for public health research |
| Verdonck-De Leeuw et al (2019)[56] | Netherlands | Longitudinal cohort case study | IDR | Advancing interdisciplinary research in head and neck cancer |
| Vohra et al (2014)[57] | Canada | Qualitative case study | IDR | Developing team-based approaches to modifiable and non-modifiable patient and provider risk factors |
| Von Lengerke (2006)[58] | Global | Commentary | IDR | Exploring critical health psychology’s capacity to contribute to public health promotion |
| Willems (2009)[59] | Senegal | Qualitative study based on focused groups | IDR | Importance of IDR in responding to HIV/AIDS vulnerability |
| Williams et al (2019)[60] | USA | Descriptive case study | MDR | Role of the multidisciplinary multisite teams in autism spectrum disorders research |
| Wright et al (2008)[61] | USA | Multimethod longitudinal case study | TDR | Opportunities and challenges of TDR on urban asthma expression in the United States. |
| Chen et al (2013)[62] | USA, Taiwan & China | Narrative analysis based on fieldwork experience | IDR | Formation and maintenance of cross-country interdisciplinary HIV research |
| Bennett et al (2012)[63] | Australia | Case study design based on focus groups and interviews | IDR | Developing interdisciplinary research practice |
| Bradley et al (2019)[64] | USA | Descriptive case study | IDR | Design and implementation of IDR in complementary and integrative health practices |
| Broder et al (2017)[65] | USA | Observation longitudinal study | MDR | Challenges and solutions sought in multidisciplinary study of youth with cleft lip and palate as well as their caregivers |
| Canning, Hird & Smith (2010)[66] | Global | Commentary | TDR | Role of epistemology in transdisciplinary public health research |
| Coyle & Mutchler (2017)[67] | Global | Commentary | CDR | Developing CDR in aging with disability |
| Ferguson et al (2019)[68] | Jamaica & USA | Descriptive case study | TDR | Use of transdisciplinary approaches in global health research |
| Garnett et al (2019)[69] | UK | Ethnographic case study | IDR | Exploring research-policy on the relationships between air pollution and human health |
| Gavens et al (2018)[70] | Europe | Interviews and discussions | IDR | Guidance on how different disciplines can collaborate to resolve complex public health problems |
| Gill et al (2017)[71] | Global | Narrative review | IDR | Importance and characteristics of Forging Alliances in Interdisciplinary Rehabilitation Research (FAIRR) logic model for conducting interdisciplinary rehabilitation research |
| Golembiewski et al (2018)[72] | USA | Analysis of pooled cross-sectional secondary survey data | IDR | Reporting of interdisciplinary  dissertations among public health doctoral graduates |
| Grigorovich et al (2019)[73] | Global | Scoping review | TDR | TDR in aging and technology |
| Hager et al (2016)[74] | USA | Mixed methods design | IDR | Fostering research skills within the context of interprofessional collaborative research practice |
| Haynes et al (2019)[75] | USA | Descriptive case study | MDR | Creating and sustaining MDR teams in worker health, safety and organisational effectiveness investigations |
| Hennessy & Walker (2011)[76] | UK | Narrative review | MDR/IDR | Developments in the promotion of MDR on ageing in the UK |
| Herzig Van Wees, Målqvist & Irwin (2019)[77] | Sweden | Commentary based on a workshop discussion | IDR | Pursuing SDGs through IDR in global health |
| Hesse-Biber (2016)[78] | Global | Keynote commentary | IDR | Problems and prospects of mixed methods healthcare IDR teams |
| Ickovics et al (2019)[79] | USA | Descriptive case study | TDR | Development and evaluation of TDR in maternal and child health |
| Linares-Pérez, (2019)[80] | Cuba | Commentary | Team science | Overcoming obstacles to team science in accelerated vaccine introduction |
| Faqua (2012)[81] | Global | Commentary | TDR | Developing understanding of TDR and related terms |
| Marrone et al (2019)[82] | USA | Case study | Multidisciplinary team science | Utility of MDR in prostate cancer tissue biomarker validation |
| O’Sullivan (2018)[83] | Ireland | Case study | Research partnerships | Developing effective research partnerships involving users |
| Raymond et al (2018)[84] | USA | Case study | IDR | Benefits and procedures of process evaluation as a tool for quality improvement in women health IDR |
| Rose, Carr & Beresford (2018)[85] | UK | Narrative review | CDR | Service user and survivor involvement in cross-disciplinary mental health research |
| Smith (2007)[86] | Global | Narrative review | TDR | Key tenants, challenges and benefits of TDR |
| Zain et al (2013)[87] | Malaysia | Descriptive case analysis | MDR | Process of setting-up and managing cross-disciplinary research centres and teams |
| Ameredes et al (2015)[88] | USA | Survey | MDR | Role of MDR in promoting career development of translational research scholars |
| Asakiewicz (2014)[89] | Global | Narrative review | Collaborative knowledge discovery | Enhancing collaborative knowledge discovery across the medical and healthcare research ecosystem |
| Asgary (2018)[90] | USA | Mixed method design | MDR | Development, implementation, and evaluation of collaborative research in global health |
| Ataman et al (2016)[91] | Global | Narrative review | CDR | Capacity building activities that strengthen CDR in global oncofertility programmes |
| Bakaki et al (2018)[92] | Global | Scoping Review | TDR | Transdisciplinary scoping review methodology in paediatric polypharmacy |
| Zainet al (2009)[93] | Malaysia | Commentary | MDR | Processes and barriers of cross-disciplinarity in oral cancer research |
| Báscolo, Yavich & de León Sánchez (2006)[94] | Argentina | Descriptive case study | CDR | Building interaction between CDR teams and decision makers |
| Basner et al (2013)[95] | Global | Narrative review | CDR | Developing effective quantitative indicators and methodologies for evaluating outcomes of CDR |
| Cars et al (2016)[96] | Sweden and China | Case study | CDR | Challenges of long international CDR |
| Domino et al (2011)[97] | USA | Case study based on participant narratives | IDR | Building IDR careers in women’s health |
| Dorsey et al (2014)[98] | USA | Case study | CDR | Sustainability of CDR centres and teams |
| Few et al (2013)[99] | Vietnam | Mixed methods design | CDR | Developing multi-layered understanding of the implications of a dynamic seasonal environment for diarrheal disease risk |
| Friedman et al (2015)[100] | USA | Qualitative case study | MDR | Achieving a learning health system through MDR |
| Guise et al (2017)[101] | USA | Mixed methods design | Interdisciplinary team science | Process, benefits, and challenges of team mentoring in interdisciplinary team science |
| Hall et al (2008)[102] | Global | Commentary | Team science | Building understanding of the processes and outcomes of team science in public health |
| Hall et al (2008)[103] | USA | Mixed methods descriptive case study | TDR | Development of new tools for assessing collaboration readiness among health researchers |
| Hall et al (2012)[104] | Global | Narrative review | TDR | Identifying the key goals and team processes of TDR |
| Koch (2010)[105] | Global | Narrative review | CDR | Emerging cross-disciplinary viewpoints on the role of technology in aged healthcare |
| Cascio et al (2016)[106] | Global | Commentary | IDR | Conceptual framework to guide future interdisciplinary investigation in autism spectrum disorder |
| Ciesielski et al (2017)[107] | Global | Narrative review | TDR | Discussion of why TDR is useful, and how it can be further promoted. |
| Feltelius et al (2015)[108] | Sweden | Narrative review | CDR | Coordinating CDR initiative to address increased incidences of narcolepsy |
| Galway et al (2016)[109] | Global | Experiential analysis | IDR | Building capacity for IDR in public health |
| Gehlert et al (2014)[110] | USA | Experiential analysis | IDR | Strategies for constructing and maintaining cross-disciplinary teams in energetics and cancer research |
| Patterson et al (2013)[111] | USA | Comparative case study design | TDR | TDR as a tool for improving the health of Americans at risk of cancer as well as cancer survivors |
| Hiatt & Breen (2008)[112] | Global | Conceptual review | TDR | Conceptual framework for organizing and disseminating TDR in cancer control |
| Marsili (2017)[113] | Global | Narrative review | CDR | CDR in environment-population health |
| Scott & Hofmeyer (2007)[114] | Global | Literature review | IDR | Influence of social and spatial contexts on interdisciplinarity in healthcare research |
| Patel et al (2007)[115] | USA | Descriptive case study | CDR | Building cross-disciplinary biorepository and bioinformatics infrastructure for cancer studies |
| Schweinhart et al (2019)[116] | USA | Commentary on preconference symposium at a women’s health conference | IDR | The emerging role of cross-disciplinary research in addressing peculiar women’s health problems |
| Miller and Leffert (2018)[117] | Global | Narrative review | CDR | Cross-disciplinary research in stroke care |
| Waage et al (2019)[118] | UK | Descriptive case study | IDR | Interdisciplinary research directed at supporting global development goals in agri-health interfaces |
| Vogel et al (2012)[119] | USA | Retrospective mixed-methods design | TDR | Potential outcomes and impacts of training in a national cancer institute-supported initiative for TDR |
| Vogel et al (2014)[120] | USA | Qualitative in-depth-interviews | TDR | Challenges, facilitating factors, strategies for success, and impacts in transdisciplinary cancer research |
| Schweinhart & Clayton (2018)[121] | USA | Narrative review | IDR | Exploring the role of IDR in addressing disparities in women health |
| Ravid et al (2017)[122] | USA | Descriptive case study | IDR | An illustrative approach to creating new faculty-driven, interdisciplinary research initiatives |
| MacGregor & Waldman (2017)[123] | Global | Narrative review | IDR | IDR on novel ways of understanding endemic zoonoses at the livestock –human interface |
| Recio et al (2016)[124] | Global | Systematic review | IDR | IDR on the quality of research work and publications |
| Guise et al (2012)[125] | USA | Mixed-methods approach using an electronic survey and in-person presentations | IDR | IDR on best practices, challenges, and lessons learned from mentoring junior faculty |
| Nagel et al (2013)[126] | USA | Descriptive study | IDR | Fostering interdisciplinary efforts to build women’s health workforce |
| Allen-Scott et al (2015)[127] | Global | Narrative review | TDR | TDR for addressing human, animal, and ecosystem interface problems |
| Calnan et al (2018)[128] | Guinea | Exploratory study based on interviews & discussion groups | IDR | IDR on Ebola virus disease survivors and other non-infected community members |
| Caduff et al (2018)[129] | Low- and middle-income countries | Literature review | IDR | Improving global cancer control through IDR |
| Salazar, Lant & Kane (2011)[130] | Global | Mixed methods based on key informant interviews and survey | IDR | Insight about which medical experts are likely to participate in IDR teams |
| Ravid et al (2013)[131] | USA | Descriptive case study | IDR | Developing interdisciplinary biomedical research |
| Miller & Bahn (2013)[132] | USA | Descriptive case study | IDR | Developing understanding of how to build IDR programmes in women health |
| Annerstedt (2010)[133] | Global | Narrative review | TDR | Transdisciplinarity as a modern research tool for investigating health problems |
| Polanco et al (2011)[134] | USA | Descriptive case study | IDR | Building an interdisciplinary team on participation of racial and ethnic minorities in  clinical trials |
| Egdell et al (2018)[135] | Global | Literature review | CDR | Developing a CDR framework on legal implications of dementia in the workplace |
| King & Gillard (2019)[136] | England | Reflexive first‐person narrative | CBPR | Ways in which CBPR enables barriers to knowledge coproduction to be overcome in mental health research |
| Perez et al (2013)[137] | USA | Descriptive case analysis | TDR | Using insight on the collaborative efforts of project managers involved in MDR to develop a tool kit for CDR project managers |

**In many cases, the design/methods and focus of the articles were not explicitly indicated. Thus, what we have captured here are inferences from the way authors presented their study objectives and conceptual/analytical frameworks.*

**References**

1. Kessel A, Green J, Pinder R, Wilkinson P, Grundy C, Lachowycz K: **Multidisciplinary research in public health: a case study of research on access to green space.** *Public health* 2009, **123:**32-38.

2. Dankwa-Mullan I, Rhee KB, Stoff DM, Pohlhaus JR, Sy FS, Stinson Jr N, Ruffin J: **Moving toward paradigm-shifting research in health disparities through translational, transformational, and transdisciplinary approaches.** *American Journal of Public Health* 2010, **100:**S19-S24.

3. Smith E, Master Z: **Best practice to order authors in multi/interdisciplinary health sciences research publications.** *Accountability in research* 2017, **24:**243-267.

4. Grönqvist H, Olsson EMG, Johansson B, Held C, Sjöström J, Norberg AL, Hovén E, Sanderman R, van Achterberg T, von Essen L: **Fifteen challenges in establishing a multidisciplinary research program on eHealth research in a university setting: A case study.** *Journal of medical Internet research* 2017, **19**.

5. Hubbard HB: **Interdisciplinary research: The role of nursing education.** *Journal of Professional Nursing* 2006, **22:**266-269.

6. Clarke D, Hawkins R, Sadler E, Harding G, Forster A, McKevitt C, Godfrey M, Monaghan J: **Interdisciplinary health research: perspectives from a process evaluation research team.** *Quality in Primary care* 2012, **20**.

7. Mazure CM, Espeland M, Douglas P, Champion V, Killien M: **Multidisciplinary women's health research: The National Centers of Excellence in Women's Health.** *Journal of women's health & gender-based medicine* 2000, **9:**717-724.

8. Canadian Academy of Health Sciences: **The Benefits and Barriers to Interdisciplinary Research in the Health Sciences in Canada.** (Sciences CAoH ed. Ontario, Canada: Canadian Academy of Health Sciences; 2005.

9. Raghunath A, Innes A: **The case for multidisciplinary research in primary care.** *Primary Health Care Research & Development* 2004, **5:**264-273.

10. Bindler RC, Richardson B, Daratha K, Wordell D: **Interdisciplinary health science research collaboration: strengths, challenges, and case example.** *Applied Nursing Research* 2012, **25:**95-100.

11. Carey GE, Smith JA: **Jack-of-all-trades, master of none: Postgraduate perspectives on interdisciplinary health research in Australia.** *BMC health services research* 2007, **7:**48.

12. Slatin C, Galizzi M, Melillo KD, Mawn B, Team PiHR: **Conducting interdisciplinary research to promote healthy and safe employment in health care: promises and pitfalls.** *Public health reports* 2004, **119:**60-72.

13. Giacomini M: **Interdisciplinarity in health services research: dreams and nightmares, maladies and remedies.** *Journal of Health Services Research & Policy* 2004, **9:**177-183.

14. Aagaard‐Hansen J: **The challenges of cross‐disciplinary research.** *Social epistemology* 2007, **21:**425-438.

15. Nair KM, Dolovich L, Brazil K, Raina P: **It's all about relationships: A qualitative study of health researchers' perspectives of conducting interdisciplinary health research.** *BMC Health Services Research* 2008, **8:**110.

16. Domino SE, Smith YR, Johnson TR: **Opportunities and challenges of interdisciplinary research career development: Implementation of a women's health research training program.** *Journal of Women's Health* 2007, **16:**256-261.

17. Urquhart R, Grunfeld E, Jackson L, Sargeant J, Porter G: **Cross-disciplinary research in cancer: an opportunity to narrow the knowledge–practice gap.** *Current Oncology* 2013, **20:**e512.

18. Laberge S, Albert M, Hodges BD: **Perspectives of clinician and biomedical scientists on interdisciplinary health research.** *Canadian Medical Association Journal* 2009, **181:**797-803.

19. Pincus HA, Keyser DJ, Schultz DJ: **RAND/Hartford initiative to build interdisciplinary geriatric health care research centers.** *Health Affairs* 2007, **26:**279-283.

20. Marts SA: **Interdisciplinary research is key to understanding sex differences: report from the Society for Women's Health Research Meeting on understanding the biology of sex differences.** *Journal of women's health & gender-based medicine* 2002, **11:**501-509.

21. Lohfeld K, Brazil L: **Understanding the collaborative experience between researchers and health care practitioners: Implications for gerontological nursing practice.** *Educational Gerontology* 2000, **26:**1-13.

22. Gebbie KM, Mason Meier B, Bakken S, Carrasquillo O, Formicola A, Aboelela SW, Glied S, Larson E: **Training for interdisciplinary health research defining the required competencies.** *Journal of Allied Health* 2008, **37:**65-70.

23. Aagaard‐Hansen J, Henry Ouma J: **Managing interdisciplinary health research–theoretical and practical aspects.** *The International journal of health planning and management* 2002, **17:**195-212.

24. Aagaard-Hansen J, Johansen MV, Riis P: **Research ethical challenges in cross-disciplinary and cross-cultural health research: the diversity of codes.** *Dan Med Bull* 2004, **51:**117-120.

25. Magnus M, Castel A: **Breaking down the siloes: developing effective multidisciplinary HIV research teams.** *AIDS and Behavior* 2016, **20:**273-280.

26. Magill-Evans J, Hodge M, Darrah J: **Establishing a transdisciplinary research team in academia.** *Journal of Allied Health* 2002, **31:**222-226.

27. Larson EL, Saiman L, Haas J, Neumann A, Lowy FD, Fatato B, Bakken S: **Perspectives on antimicrobial resistance: Establishing an interdisciplinary research approach.** *American journal of infection control* 2005, **33:**410-418.

28. Urquhart R, Porter GA, Grunfeld E: **Reflections on knowledge brokering within a multidisciplinary research team.** *Journal of Continuing Education in the Health Professions* 2011, **31:**283-290.

29. Albrecht TL, Kuerer H, Ruckdeschel JC, Mendez J, Harper F: **Transdisciplinary studies of surgical oncology trial accrual: A National Institutes of Health/National Cancer Institute Roadmap-affiliated project update.** *Cancer* 2006, **107:**171-174.

30. An G, Hunt CA, Clermont G, Neugebauer E, Vodovotz Y: **Challenges and rewards on the road to translational systems biology in acute illness: four case reports from interdisciplinary teams.** *Journal of Critical Care* 2007, **22:**169-175.

31. Fletcher S, Whiting C, Boaz A, Reeves S: **Exploring factors related to the translation of collaborative research learning experiences into clinical practice: Opportunities and tensions.** *Journal of Interprofessional Care* 2017, **31:**543-545.

32. Cooper LA, Boulware LE, Miller 3rd ER, Golden SH, Carson KA, Noronha G, Huizinga MM, Roter DL, Yeh HC, Bone LR, et al: **Creating a transdisciplinary research center to reduce cardiovascular health disparities in Baltimore, Maryland: lessons learned.** *American journal of public health* 2013, **103:**e26-38.

33. Corbett C, Costa L, Balas M, Burke W, Feroli E, Daratha K: **Facilitators and challenges to conducting interdisciplinary research.** *Medical Care* 2013, **51:**S23-S31.

34. Denne SC, Sajdyk T, Sorkness CA, Drezner MK, Shekhar A: **Utilizing pilot funding and other incentives to stimulate interdisciplinary research.** *Translational Research in Biomedicine* 2013, **3:**63-73.

35. Goldsack JC, Michalec B, Cipolle M, Sonnad SS: **Remote and interdisciplinary research in surgical knowledge production.** *Journal of Surgical Research* 2016, **202:**139-146.

36. Grey M, Connolly CA: **"Coming together, keeping together, working together": Interdisciplinary to transdisciplinary research and nursing.** *Nursing Outlook* 2008, **56:**102-107.

37. Grigorovich A, Pia K, Sixsmith J: **Transdisciplinary research and innovation: Improving development and uptake of technologies to support persons with dementia.** *Alzheimer's and Dementia* 2016, **12 (7 Supplement):**P153-P154.

38. Harper GW, Neubauer LC, Bangi AK, Francisco VT: **Transdisciplinary research and evaluation for community health initiatives.** *Health promotion practice* 2008, **9:**328-337.

39. Harris JK, Beatty KE, Lecy JD, Cyr JM, Shapiro IRM: **Mapping the multidisciplinary field of public health services and systems research.** *American Journal of Preventive Medicine* 2011, **41:**105-111.

40. Hebert JR, Brandt HM, Armstead CA, Adams SA, Steck SE: **Interdisciplinary, translational, and community-based participatory research: Finding a common language to improve cancer research.** *Cancer Epidemiology Biomarkers and Prevention* 2009, **18:**1213-1217.

41. Hills H, Richards T: **Modeling interdisciplinary research to advance behavioral health care.** *The journal of behavioral health services & research* 2014, **41:**3-7.

42. Holmes JH, Lehman A, Hade E, Ferketich AK, Gehlert S, Rauscher GH, Abrams J, Bird CE: **Challenges for Multilevel Health Disparities Research in a Transdisciplinary Environment.** *American Journal of Preventive Medicine* 2008, **35:**S182-S192.

43. Kessel F, Rosenfield PL: **Toward Transdisciplinary Research. Historical and Contemporary Perspectives.** *American Journal of Preventive Medicine* 2008, **35:**S225-S234.

44. Kestenbaum A, James J, Morgan S, Shields M, Hocker W, Rabow M, Dunn LB: **"Taking your place at the table": An autoethnographic study of chaplains' participation on an interdisciplinary research team.** *BMC Palliative Care* 2015**:**1-10.

45. Iida EE, Springer JF, Pecora PJ, Bandstra ES, Edwards MC, Basen MM: **The SESS multisite collaborative research initiative: establishing common ground.** *Child & Family Social Work* 2005, **10:**217-228.

46. Kobus K, Mermelstein R: **Bridging basic and clinical science with policy studies: The Partners with Transdisciplinary Tobacco Use Research Centers experience.** *Nicotine and Tobacco Research* 2009, **11:**467-474.

47. Lakhani J, Benzies K, Hayden KA: **Attributes of interdisciplinary research teams: a comprehensive review of the literature.** *Clinical and investigative medicine* 2012, **Medecine clinique et experimentale. 35:**E226.

48. Mabry PL, Olster DH, Morgan GD, Abrams DB: **Interdisciplinarity and Systems Science to Improve Population Health. A View from the NIH Office of Behavioral and Social Sciences Research.** *American Journal of Preventive Medicine* 2008, **35:**S211-S224.

49. Neill KM: **A holistic interdisciplinary health care research model.** *Holistic nursing practice* 1999, **13:**54-60.

50. O'Cathain A, Murphy E, Nicholl J: **Multidisciplinary, interdisciplinary, or dysfunctional? Team working in mixed-methods research.** *Qualitative Health Research* 2008, **18:**1574-1585.

51. Person H, Smalls-Mantey AR, Ayeni O, Hernandez-Saurez D, Benn EKT, Bagiella E, Gabrilove JL: **Fostering cross-disciplinary research: Lessons learned from STTEP-UP.** *Journal of Clinical and Translational Science* 2018**:**56.

52. Ranford J, Asiello J, Cloutier A, Cortina K, Thorne H, Erler KS, Frazier N, Sadlak C, Rude A, Lin DJ: **Interdisciplinary Stroke Recovery Research: The Perspective of Occupational Therapists in Acute Care.** *Frontiers in Neurology* 2019, **10 (no pagination)**.

53. Schultz D, Keyser D, Pincus HA: **Developing interdisciplinary centers in aging: Learning from the RAND/Hartford building interdisciplinary geriatric health care research centers initiative.** *Academic Medicine* 2011, **86:**1318-1324.

54. Tran BX, Nathan KI, Phan HT, Hall BJ, Vu GT, Vu LG, Pham HQ, Latkin CA, Ho CSH, Ho RCM: **Evolution of Interdisciplinary Landscapes of HIV/Acquired Immune Deficiency Syndromes Studies from 1983 to 2017: Results from the Global Analysis for Policy in Research (GAPRESEARCH).** *AIDS reviews* 2019, **21**.

55. Turnbull ER, Pineo H, Aldridge RW: **Improving the health of the public: a transdisciplinary research study.** *The Lancet* 2019, **394 (Supplement 2):**S93.

56. Verdonck-de Leeuw I, Jansen F, Brakenhoff RH, Langendijk JA, Takes R, Terhaard CHJ, de Jong RJB, Smit JH, Leemans CR: **Advancing interdisciplinary research in head and neck cancer through a multicenter longitudinal prospective cohort study: the NETherlands QUality of life and BIomedical Cohort (NET-QUBIC) data warehouse and biobank.** *BMC Cancer* 2019, **19**.

57. Vohra S, Kawchuk GN, Boon H, Caulfield T, Pohlman KA, O'Beirne M: **SafetyNET: An interdisciplinary research program to support a safety culture for spinal manipulation therapy.** *European Journal of Integrative Medicine* 2014, **6:**473-477.

58. Von Lengerke T: **Public health is an interdiscipline, and about wholes and parts: Indeed, critical health psychology needs to join forces.** *Journal of Health Psychology* 2006, **11:**395-399.

59. Willems R: **The importance of interdisciplinary collaborative research in responding to HIV/AIDS vulnerability in rural Senegal.** *African Journal of AIDS Research* 2009, **8:**433-442.

60. Williams ME, Capal JK, Byars AW, Kissinger R, Hanson E, Wu JY, Bebin EM, Krueger D, Pearson DA, Murray DS, et al: **Impacting Development in Infants With Tuberous Sclerosis Complex: Multidisciplinary Research Collaboration.** *American Psychologist* 2019, **74:**356-367.

61. Wright RJ, Suglia SF, Levy J, Fortun K, Shields A, Subramanian SV, Wright R: **Transdisciplinary research strategies for understanding socially patterned disease: The Asthma Coalition on Community, Environment, and Social Stress (ACCESS) project as a case study.** *Ciencia e Saude Coletiva* 2008, **13:**1729-1742.

62. Chen WT, Shiu CS, Simoni JM, Chuang P, Zhao H, Bao M, Lu H: **Challenges of cross-cultural research: lessons from a U.S.-Asia HIV collaboration.** *Nurs Outlook* 2013, **61:**145-152.

63. Bennett E, Hauck Y, Bindahneem S, Banham V, Owens M, Priddis LE, Wells G, Sinclair W, Shields L: **The development of an interdisciplinary research agenda at Ngala: an innovative case study.** *Neonatal, Paediatric & Child Health Nursing* 2012, **15:**20-25.

64. Bradley R, Booth-LaForce C, Hanes D, Scott C, Sherman KJ, Lin YS, Zwickey H: **Design of a Multidisciplinary Training Program in Complementary and Integrative Health Clinical Research: Building Research Across Interdisciplinary Gaps.** *Journal of Alternative & Complementary Medicine* 2019, **25:**509-516.

65. Broder HL, Crerand CE, Ruff RR, Peshansky A, Sarwer DB, Sischo L: **Challenges in conducting multicentre, multidisciplinary, longitudinal studies in children with chronic conditions.** *Community dentistry and oral epidemiology* 2017, **45:**317-322.

66. Canning CG, Hird M, Smith G: **'The pitfalls of the 'add-and-stir' approach to transdisciplinary public health research'.** *Critical Public Health* 2010, **20:**145-155.

67. Coyle CE, Mutchler JE: **Aging With Disability: Advancement of a Cross-Disciplinary Research Network.** *Research on Aging* 2017, **39:**683-692.

68. Ferguson GM, Fiese BH, Nelson MR, Meeks Gardner JM: **Transdisciplinary Team Science for Global Health: Case Study of the JUS Media? Programme.** *American Psychologist* 2019, **74:**725-739.

69. Garnett E, Green J, Chalabi Z, Wilkinson P: **Materialising links between air pollution and health: How societal impact was achieved in an interdisciplinary project.** *Health (London, England : 1997)* 2019, **23:**234-252.

70. Gavens L, Holmes J, Buhringer G, McLeod J, Neumann M, Lingford-Hughes A, Hock ES, Meier PS: **Interdisciplinary working in public health research: a proposed good practice checklist.** *Journal of public health (Oxford, England)* 2018, **40:**175-182.

71. Gill SV, Khetani MA, Yinusa-Nyahkoon L, McManus B, Gardiner PM, Tickle-Degnen L: **Forging alliances in interdisciplinary rehabilitation research (FAIRR): A logic model.** *Am J Phys Med Rehabil* 2017, **96:**479-486.

72. Golembiewski EH, Holmes AM, Jackson JR, Brown-Podgorski BL, Menachemi N: **Interdisciplinary Dissertation Research Among Public Health Doctoral Trainees, 2003-2015.** *Public Health Rep* 2018, **133:**182-190.

73. Grigorovich A, Fang ML, Sixsmith J, Kontos P: **Defining and evaluating transdisciplinary research: implications for aging and technology.** *Disability & Rehabilitation: Assistive Technology* 2019, **14:**533-542.

74. Hager K, St Hill C, Prunuske J, Swanoski M, Anderson G, Lutfiyya MN: **Development of an interprofessional and interdisciplinary collaborative research practice for clinical faculty.** *Journal of interprofessional care* 2016, **30:**265-267.

75. Haynes NJ, Vandenberg RJ, DeJoy DM, Wilson MG, Padilla HM, Zuercher HS, Robertson MM: **The Workplace Health Group: A Case Study of 20 Years of Multidisciplinary Research.** *American Psychologist* 2019, **74:**380-393.

76. Hennessy CH, Walker A: **Promoting multi-disciplinary and inter-disciplinary ageing research in the United Kingdom.** *Ageing & Society* 2011, **31:**52-69.

77. Herzig Van Wees SL, Malqvist M, Irwin R: **Achieving the SDGs through interdisciplinary research in global health.** *Scandinavian journal of public health* 2019, **47:**793-795.

78. Hesse-Biber S: **Doing Interdisciplinary Mixed Methods Health Care Research: Working the Boundaries, Tensions, and Synergistic Potential of Team-Based Research.** *Qualitative health research* 2016, **26:**649-658.

79. Ickovics JR, Lewis JB, Cunningham SD, Thomas J, Magriples U: **Transforming prenatal care: Multidisciplinary team science improves a broad range of maternal-child outcomes.** *The American psychologist* 2019, **74:**343-355.

80. Linares-Pérez N: **Team Science and Accelerated Vaccine Introduction in Cuba: A View from the Pneumococcal Project...Translated from Spanish and reprinted with permission from Revista Cubana de Salud Publica Vol 44 No 2, Apr–Jun 2018.** *MEDICC Review* 2019, **21:**37-38.

81. Fuqua J: **Toward a Better Understanding of the Definition of Transdisciplinary Scientific Collaboration.** *Californian Journal of Health Promotion* 2012, **10:**vi-xiii.

82. Marrone MT, Joshu CE, Peskoe SB, De Marzo AM, Heaphy CM, Lupold SE, Meeker AK, Platz EA: **Adding the Team into T1 Translational Research: A Case Study of Multidisciplinary Team Science in the Evaluation of Biomarkers of Prostate Cancer Risk and Prognosis.** *Clinical Chemistry* 2019, **65:**189-198.

83. O'Sullivan R: **Research partnerships – embracing user involvement: practical considerations and reflections.** *Quality in Ageing & Older Adults* 2018, **19:**220-231.

84. Raymond NC, Wyman JF, Dighe S, Harwood EM, Hang M: **Process Evaluation for Improving K12 Program Effectiveness: Case Study of a National Institutes of Health Building Interdisciplinary Research Careers in Women's Health Research Career Development Program.** *Journal of Women's Health (15409996)* 2018, **27:**775-781.

85. Rose D, Carr S, Beresford P: **'Widening cross-disciplinary research for mental health': what is missing from the Research Councils UK mental health agenda?** *Disability & Society* 2018, **33:**476-481.

86. Smith PM: **A transdisciplinary approach to research on work and health: what is it, what could it contribute, and what are the challenges?** *Critical Public Health* 2007, **17:**159-169.

87. Zain RB, Athirajan V, Ghani WMN, Razak IA, Raja Latifah RJ, Ismail SM, Sallam AA, Bustam AZ, Rahman ZAA, Hussien A, et al: **An oral cancer biobank initiative: A platform for multidisciplinary research in a developing country.** *Cell and Tissue Banking* 2013, **14:**45-52.

88. Ameredes BT, Hellmich MR, Cestone CM, Wooten KC, Ottenbacher KJ, Chonmaitree T, Anderson KE, Brasier AR: **The Multidisciplinary Translational Team (MTT) Model for Training and Development of Translational Research Investigators.** *Clin Transl Sci* 2015, **8:**533-541.

89. Asakiewicz C: **Translational Research 2.0: a framework for accelerating collaborative discovery.** *Per Med* 2014, **11:**351-358.

90. Asgary R: **A Collaborative Multidisciplinary and Without-Walls Research Curriculum in Global Health.** *Am J Trop Med Hyg* 2018, **99:**1283-1290.

91. Ataman LM, Rodrigues JK, Marinho RM, Caetano JP, Chehin MB, Alves da Motta EL, Serafini P, Suzuki N, Furui T, Takae S, et al: **Creating a Global Community of Practice for Oncofertility.** *J Glob Oncol* 2016, **2:**83-96.

92. Bakaki PM, Staley J, Liu R, Dawson N, Golchin N, Horace A, Johnson H, Waldron J, Winterstein A, Kleinman LC, Bolen SD: **A transdisciplinary team approach to scoping reviews: the case of pediatric polypharmacy.** *BMC Med Res Methodol* 2018, **18:**102.

93. Zain RB, Ghani WMN, Razak IA, Latifah RJR, Samsuddin AR, Cheong SC, Abdullah N, Ismail AR, Hussaini HB, Talib NA, Jallaludin A: **Building partnership in oral cancer research in a developing country - Processes and barriers.** *Asian Pacific Journal of Cancer Prevention* 2009, **10:**513-518.

94. Bascolo E, Yavich N, Sanchez de Leon A: **Interaction between researchers and decision-makers: a case study.** *Cad Saude Publica* 2006, **22 Suppl:**S47-56.

95. Basner JE, Theisz KI, Jensen US, Jones CD, Ponomarev I, Sulima P, Jo K, Eljanne M, Espey MG, Franca-Koh J, et al: **Measuring the evolution and output of cross-disciplinary collaborations within the NCI Physical Sciences-Oncology Centers Network.** *Res Eval* 2013, **22:**285-297.

96. Cars O, Xiao Y, Stalsby Lundborg C, Nilsson LE, Shen J, Sun Q, Bi Z, Borjesson S, Greko C, Wang Y, et al: **Building bridges to operationalise one health - A Sino-Swedish collaboration to tackle antibiotic resistance.** *One Health* 2016, **2:**139-143.

97. Domino SE, Bodurtha J, Nagel JD: **Interdisciplinary Research Career Development: Building Interdisciplinary Research Careers in Women's Health Program Best Practices.** *J Womens Health (Larchmt)* 2011, **20:**1587-1601.

98. Dorsey SG, Schiffman R, Redeker NS, Heitkemper M, McCloskey DJ, Weglicki LS, Grady PA: **National Institute of Nursing Research Centers of Excellence: a logic model for sustainability, leveraging resources, and collaboration to accelerate cross-disciplinary science.** *Nurs Outlook* 2014, **62:**384-393.

99. Few R, Lake I, Hunter PR, Tran PG: **Seasonality, disease and behavior: using multiple methods to explore socio-environmental health risks in the Mekong Delta.** *Soc Sci Med* 2013, **80:**1-9.

100. Friedman C, Rubin J, Brown J, Buntin M, Corn M, Etheredge L, Gunter C, Musen M, Platt R, Stead W, et al: **Toward a science of learning systems: a research agenda for the high-functioning Learning Health System.** *J Am Med Inform Assoc* 2015, **22:**43-50.

101. Guise JM, Geller S, Regensteiner JG, Raymond N, Nagel J: **Team Mentoring for Interdisciplinary Team Science: Lessons from K12 Scholars and Directors.** *Acad Med* 2017, **92:**214-221.

102. Hall KL, Feng AX, Moser RP, Stokols D, Taylor BK: **Moving the Science of Team Science Forward: Collaboration and Creativity.** *Am J Prev Med* 2008, **35:**S243-249.

103. Hall KL, Stokols D, Moser RP, Taylor BK, Thornquist MD, Nebeling LC, Ehret CC, Barnett MJ, McTiernan A, Berger NA, et al: **The collaboration readiness of transdisciplinary research teams and centers findings from the National Cancer Institute's TREC Year-One evaluation study.** *Am J Prev Med* 2008, **35:**S161-172.

104. Hall KL, Vogel AL, Stipelman BA, Stokols D, Morgan G, Gehlert S: **A four-phase model of transdisciplinary team-based research: goals, team processes, and strategies.** *Transl Behav Med* 2012, **2:**415-430.

105. Koch S: **Healthy ageing supported by technology--a cross-disciplinary research challenge.** *Inform Health Soc Care* 2010, **35:**81-91.

106. Cascio CJ, Woynaroski T, Baranek GT, Wallace MT: **Toward an interdisciplinary approach to understanding sensory function in autism spectrum disorder.** *Autism Res* 2016, **9:**920-925.

107. Ciesielski TH, Aldrich MC, Marsit CJ, Hiatt RA, Williams SM: **Transdisciplinary approaches enhance the production of translational knowledge.** *Transl Res* 2017, **182:**123-134.

108. Feltelius N, Persson I, Ahlqvist-Rastad J, Andersson M, Arnheim-Dahlstrom L, Bergman P, Granath F, Adori C, Hokfelt T, Kuhlmann-Berenzon S, et al: **A coordinated cross-disciplinary research initiative to address an increased incidence of narcolepsy following the 2009-2010 Pandemrix vaccination programme in Sweden.** *J Intern Med* 2015, **278:**335-353.

109. Galway LP, Parkes MW, Allen D, Takaro TK: **Building Interdisciplinary Research Capacity: a Key Challenge for Ecological Approaches in Public Health.** *AIMS Public Health* 2016, **3:**389-406.

110. Gehlert S, Hall K, Vogel A, Hohl S, Hartman S, Nebeling L, Redline S, Schmitz K, Thornquist M, Patterson R, Thompson B: **Advancing Transdisciplinary Research: The Transdisciplinary Research on Energetics and Cancer Initiative.** *J Transl Med Epidemiol* 2014, **2:**1032.

111. Patterson RE, Colditz GA, Hu FB, Schmitz KH, Ahima RS, Brownson RC, Carson KR, Chavarro JE, Chodosh LA, Gehlert S, et al: **The 2011-2016 Transdisciplinary Research on Energetics and Cancer (TREC) Initiative: Rationale and Design.** *Cancer Causes and Control* 2013, **24:**695-704.

112. Hiatt RA, Breen N: **The social determinants of cancer: a challenge for transdisciplinary science.** *Am J Prev Med* 2008, **35:**S141-150.

113. Marsili D: **A cross-disciplinary approach to global environmental health: the case of contaminated sites.** *Ann Ist Super Sanita* 2016, **52:**516-523.

114. Scott CM, Hofmeyer AT: **Acknowledging complexity: critically analyzing context to understand interdisciplinary research.** *J Interprof Care* 2007, **21:**491-501.

115. Patel AA, Gilbertson JR, Showe LC, London JW, Ross E, Ochs MF, Carver J, Lazarus A, Parwani AV, Dhir R, et al: **A novel cross-disciplinary multi-institute approach to translational cancer research: lessons learned from Pennsylvania Cancer Alliance Bioinformatics Consortium (PCABC).** *Cancer Inform* 2007, **3:**255-274.

116. Schweinhart A, Cargill V, Brady K, Hall K, Spencer E, Clayton J: **Addressing Health Challenges of Women Across the Life Course: Summary of the Women's Health 2018 Preconference Symposium.** *Journal of Women's Health* 2019, **28:**741-746.

117. Miller EC, Leffert L: **Building Cross-Disciplinary Research Collaborations.** *Stroke* 2018, **49:**e43-e45.

118. Waage J, Cornelsen L, Dangour AD, Green R, Häsler B, Hull E, Johnston D, Kadiyala S, Lock K, Shankar B, et al: **Integrating Agriculture and Health Research for Development: LCIRAH as an Interdisciplinary Programme to Address a Global Challenge.** *Glob Chall* 2019, **3**.

119. Vogel AL, Feng A, Oh A, Hall KL, Stipelman BA, Stokols D, Okamoto J, Perna FM, Moser R, Nebeling L: **Influence of a National Cancer Institute transdisciplinary research and training initiative on trainees' transdisciplinary research competencies and scholarly productivity.** *Translational Behavioral Medicine* 2012, **2:**459-468.

120. Vogel AL, Stipelman BA, Hall KL, Nebeling L, Stokols D, Spruijt-Metz D: **Pioneering the Transdisciplinary Team Science Approach: Lessons Learned from National Cancer Institute Grantees.** *J Transl Med Epidemiol* 2014, **2**.

121. Schweinhart A, Clayton J: **Reversing the Trends toward Shorter Lives and Poorer Health for US Women: A Call for Innovative Interdisciplinary Research.** *International journal of environmental research and public health* 2018, **15:**1796.

122. Ravid K, Seta F, Center D, Waters G, Coleman D: **Catalyzing Interdisciplinary Research and Training: Initial Outcomes and Evolution of the Affinity Research Collaboratives Model.** *Academic medicine : journal of the Association of American Medical Colleges* 2017, **92:**1399-1405.

123. MacGregor H, Waldman L: **Views from many worlds: unsettling categories in interdisciplinary research on endemic zoonotic diseases.** *Philos Trans R Soc Lond B Biol Sci* 2017, **372**.

124. Recio GM, Garcia-Hernandez L, Luque RM, Salas-Morera LJBeo: **The role of interdisciplinary research team in the impact of health apps in health and computer science publications: a systematic review.** 2016, **15:**77.

125. Guise JM, Nagel JD, Regensteiner JG, Freund KM, Silliman R, Sherman BJ, Leppert PC, Goldstein JM, Kaiser UB, Miller VM, et al: **Best Practices and pearls in interdisciplinary mentoring from building interdisciplinary research careers in women's health directors.** *Journal of Women's Health* 2012, **21:**1114-1127.

126. Nagel JD, Koch A, Guimond JM, Glavin S, Geller S: **Building the Women's Health Research Workforce: Fostering Interdisciplinary Research Approaches in Women's Health.** *Global Advances In Health and Medicine* 2013, **2:**24-29.

127. Allen-Scott LK, Buntain B, Hatfield JM, Meisser A, Thomas CJ: **Academic Institutions and One Health: Building Capacity for Transdisciplinary Research Approaches to Address Complex Health Issues at the Animal-Human-Ecosystem Interface.** *Academic medicine : journal of the Association of American Medical Colleges* 2015, **90:**866-871.

128. Calnan M, Gadsby EW, Kondé MK, Diallo A, Rossman JS: **The Response to and Impact of the Ebola Epidemic: Towards an Agenda for Interdisciplinary Research.** *Int J Health Policy Manag* 2018, **7:**402-411.

129. Caduff C, Skelton M, Banerjee D, Djordjevic D, Mika M, Mueller L, Sivaramakrishnan K, Van Hollen C: **Analysis of Social Science Research Into Cancer Care in Low- and Middle-Income Countries: Improving Global Cancer Control Through Greater Interdisciplinary Research.** *J Glob Oncol* 2018, **4**.

130. Salazar M, Lant T, Kane A: **To Join or Not to Join: An Investigation of Individual Facilitators and Inhibitors of Medical Faculty Participation in Interdisciplinary Research Teams.** *Clinical and Translational Science* 2011, **4:**274-278.

131. Ravid K, Faux R, Corkey B, Coleman D: **Building interdisciplinary biomedical research using novel collaboratives.** *Academic Medicine* 2013, **88:**179-184.

132. Miller VM, Bahn RS: **Mentoring Interdisciplinary Research Teams for the Study of Sex and Gender Differences in Health and Disease.** *Global Advances In Health and Medicine* 2013, **2:**109-112.

133. Annerstedt M: **Transdisciplinarity as an inference technique to achieve a better understanding in the health and environmental sciences.** *International Journal of Environmental Research and Public Health* 2010, **7:**2692-2707.

134. Polanco FR, Dominguez DC, Grady C, Stoll P, Ramos C, Mican JM, Miranda-Acevedo R, Morgan M, Aizvera J, Purdie LJJotAoNiAC: **Conducting HIV research in racial and ethnic minority communities: building a successful interdisciplinary research team.** 2011, **22:**388-396.

135. Egdell V, Stavert J, McGregor R: **The legal implications of dementia in the workplace: establishing a cross-disciplinary research agenda.** *Ageing & Society* 2018, **38:**2181-2196.

136. King C, Gillard S: **Bringing together coproduction and community participatory research approaches: Using first person reflective narrative to explore coproduction and community involvement in mental health research.** *Health Expectations* 2019, **22:**701-708.

137. Perez NA, Weathers B, Willis M, Mendez J: **Collaboration Across Eight Research Centers: Unanticipated Benefits and Outcomes for Project Managers.** *Population Health Management* 2013, **16:**46-52.
